# Bacteriocins to Thwart Bacterial Resistance in Gram Negative Bacteria

**DOI:** 10.3389/fmicb.2020.586433

**Published:** 2020-11-09

**Authors:** Soufiane Telhig, Laila Ben Said, Séverine Zirah, Ismail Fliss, Sylvie Rebuffat

**Affiliations:** ^1^Institute of Nutrition and Functional Foods, Université Laval, Québec, QC, Canada; ^2^Laboratory Molecules of Communication and Adaptation of Microorganisms, Muséum National d’Histoire Naturelle, Centre National de la Recherche Scientifique, Paris, France

**Keywords:** bacteriocins, microcins, antibiotics, resistance, Gram-negative bacteria, enterobacteria

## Abstract

An overuse of antibiotics both in human and animal health and as growth promoters in farming practices has increased the prevalence of antibiotic resistance in bacteria. Antibiotic resistant and multi-resistant bacteria are now considered a major and increasing threat by national health agencies, making the need for novel strategies to fight bugs and super bugs a first priority. In particular, Gram-negative bacteria are responsible for a high proportion of nosocomial infections attributable for a large part to *Enterobacteriaceae*, such as pathogenic *Escherichia coli*, *Klebsiella pneumoniae*, and *Pseudomonas aeruginosa*. To cope with their highly competitive environments, bacteria have evolved various adaptive strategies, among which the production of narrow spectrum antimicrobial peptides called bacteriocins and specifically microcins in Gram-negative bacteria. They are produced as precursor peptides that further undergo proteolytic cleavage and in many cases more or less complex posttranslational modifications, which contribute to improve their stability and efficiency. Many have a high stability in the gastrointestinal tract where they can target a single pathogen whilst only slightly perturbing the gut microbiota. Several microcins and antibiotics can bind to similar bacterial receptors and use similar pathways to cross the double-membrane of Gram-negative bacteria and reach their intracellular targets, which they also can share. Consequently, bacteria may use common mechanisms of resistance against microcins and antibiotics. This review describes both unmodified and modified microcins [lasso peptides, siderophore peptides, nucleotide peptides, linear azole(in)e-containing peptides], highlighting their potential as weapons to thwart bacterial resistance in Gram-negative pathogens and discusses the possibility of cross-resistance and co-resistance occurrence between antibiotics and microcins in Gram-negative bacteria.

## Introduction

Since their discovery antibiotics have been routinely used in human medicine and in livestock production as therapeutic agents or growth promoters. Use of antibiotics for livestock greatly exceeds that of uses for humans, with approximately 70–80 percent of total consumption (Van Boeckel et al., 2017). Furthermore, the global use of antibiotics would rise by 67% by 2030 in high-income countries and nearly double in Brazil, the Russian Federation, India, China and South Africa (Van Boeckel et al., 2015). According to the World Health Organization (World Health Organization [WHO], 2017) the overuse and misuse of antibiotics in human and animal, as well as the intrinsic capacity of antibiotics to induce broad spectrum killing (Wester et al., 2002) has led to the emergence of multidrug-resistant bacteria (MDR) that are rapidly increasing worldwide and have now become a serious public health problem. In 2016, the United Nations General Assembly recognized the use of antibiotics in the livestock sector as one of the primary causes of antimicrobial resistance (AMR) (Van Boeckel et al., 2017). Moreover, it has been shown that farm animal and human microbiota are reservoirs for AMR (Gibson et al., 2016; Pärnänen et al., 2018; Brown et al., 2019; Sun et al., 2020). Currently, AMR is already killing 700,000 people a year, and it is predicted to cause 10 million deaths per year by 2050 with a cumulative cost of US$ 100 trillion (de Kraker et al., 2016). According to the Centers for Disease Control and Prevention (CDC) AMR challenge, *Enterobacteriaceae*, including *Escherichia coli, Shigella*, *Salmonella*, and *Klebsiella* spp. amongst others, present a serious and/or urgent threat to world health. Indeed, as Gram-negative bacteria, *Enterobacteriaceae* are notorious for their capacity to resist antimicrobial therapy (Hawkey, 2015; Li et al., 2015; Zowawi et al., 2015). Furthermore, even though *Enterobacteriaceae* represent only a small percentage of the host microbiota and are not all pathogens, they are still responsible for important morbidity (Doi et al., 2017; MacVane, 2017), making them an important target for new drug development.

The AMR crisis is exacerbated by the fact that resistances are emerging and disseminating faster than the development of new drugs. Indeed, over the past three decades the number of developed and approved antibiotics has more than halved (Ventola, 2015), leading to an increasing demand for new antimicrobial agents or strategies. Genetically modified phages, antibacterial modified oligonucleotides, inhibitors of bacterial virulence and CRISPR-Cas9 strategy are also discussed for extrapolating them to the field of antimicrobial therapeutics (Dickey et al., 2017; Ghosh et al., 2019). Meanwhile, other promising strategies, such as probiotics, lysins and antimicrobial peptides are in various stages of development (Ghosh et al., 2019). Globally, although several alternatives exist in nature, the challenge still remains to demonstrate their efficacy and their use in human and animal.

Bacteriocins form a large family of antimicrobial peptides (AMP) produced by bacteria (Klaenhammer, 1988). Their biological characteristics and activities have been deeply described in a new web-accessible database named BACTIBASE, which is freely available at the http://bactibase.pfba-lab.org web-based platform. Bacteriocins can be either unmodified or posttranslationally modified peptides, the latter thus belonging to the large family of ribosomally synthesized and posttranslationally modified peptides (RiPPs) (Arnison et al., 2013; Montalbán-López et al., 2020). Known as inhibitors of pathogens *in vitro*, many bacteriocins have a high specific activity against clinical strains including antibiotic-resistant ones (Cotter et al., 2013). Their effectiveness as inhibitors of pathogenic and spoilage microorganisms has been largely explored (Davies et al., 1997; Deegan et al., 2006). It is thus widely believed that some could be usable for therapeutic purposes and as an alternative to conventional antibiotics (Snyder and Worobo, 2014; Egan et al., 2017).

Bacteriocins produced by enterobacteria are called microcins (Baquero and Moreno, 1984). They form a restricted and underexplored group of bacteriocins compared to the hundreds members of those from lactic acid bacteria, with only some twenty members identified so far, among which only around fifteen have been more deeply characterized (Table 1 and Supplementary Figure S1). Microcins are less than 10 kDa modified or unmodified peptides (Rebuffat, 2012) having key ecological functions, and particularly a role in microbial competitions (Baquero et al., 2019; Li and Rebuffat, 2020). They have potent activity with minimum inhibitory concentrations (MIC) ranging in the nanomolar to micromolar range and narrow spectra of antimicrobial activity directed essentially against Gram-negative bacterial congeners (Rebuffat, 2012; Baquero et al., 2019). To exert their crucial roles in competition, microcins share a common strategy to penetrate into their bacterial targets. They piratize nutrient uptake pathways of phylogenetically close bacteria vying for the same resources. The iron import pathways is the most frequently attacked (Rebuffat, 2012). When inside bacteria, microcins interfere and perturb a variety of bacterial mechanisms, such as transcription (Adelman et al., 2004), translation (Metlitskaya et al., 2006), DNA structure (Vizán et al., 1991), mannose transport (Bieler et al., 2006), energy production (Trujillo et al., 2001; Zhao et al., 2015), or the cell envelope function (Destoumieux-Garzón et al., 2003; Gerard et al., 2005; Zhao et al., 2015). Due to their specific characteristics and complex mechanisms of action, microcins are viewed as a possible alternative to conventional antibiotics, helping with the immediate AMR problem (Cotter et al., 2013; Mills et al., 2017; Lu et al., 2019; Palmer et al., 2020). Because of their narrow spectrum of inhibition, they would potentially have less side effects than antibiotics, allowing preservation of the microbiota diversity and minimizing the risk of resistance dissemination.

**TABLE 1:** Structural characterization of microcins assembled into posttranslationally modified microcins (classes I and IIb) and unmodified microcins (class IIa) that contain or not disulfide bridges.

Class	Microcin	MM^(a)^ (Da)	PTMs/disulfide bonds	Structure	Producing organism	References
Class I (modified)	McC	1177	Peptidyladenylate with the C-terminal Asp^7^ linked to AMP via a phosphoramidate linkage and bearing an aminopropyl on the phosphate	Nucleotide peptide	*E. coli*	Guijarro et al., 1995
	MccJ25	2107	Macrolactam ring between Gly^1^ and Glu^8^ threaded by the Tyr^9^- Gly^21^ tail locked inside by Phe^19^ and Tyr^20^ side chains (lasso topology)	Lasso peptide	*E. coli*	Rosengren et al., 2003
	MccB17	3093	Gly^39^Ser^40^Cys^41^ and Gly^54^Cys^55^Ser^56^ motifs modified to oxazole-thiazole and thiazole-oxazole heterocycles	Linear azol(in)e-containing peptide (LAP)^(b)^	*E. coli*	Li et al., 1996
Class IIa (unmodified)	MccV	8734	1 disulfide bond (Cys^76^ – Cys^87^)	Unmodified peptide	*E. coli*	Fath et al., 1994
	MccL	8884	2 disulfide bonds (Cys^29^ – Cys^33^; Cys^78^ – Cys^89^)	Unmodified peptide	*E. coli* LR05	Pons et al., 2004
	MccS^(c)^	9746	2 putative disulfide bonds (Cys^57^, Cys^90^, Cys^109^, Cys^118^)	Unmodified peptide	*E. coli* G3/10	Zschüttig et al., 2012
	MccPDI^(c)^ MccN/24	9953 7222	2 putative disulfide bonds (Cys^57^, Cys^90^, Cys^109^, Cys^118^) with Cys^57^-Cys ^90^ bond required for activity No disulfide bond (no Cys residue)	Unmodified peptide Unmodified peptide	*E. coli* 25 Uropathogenic *E. coli*	Eberhart et al., 2012 Kaur et al., 2016
Class IIb	MccE492	7887^(d)^ 8718^(e)^	Linear trimer of N-2,3-(dihydroxybenzoyl)-L-serine (DHBS) anchored at the C-terminal Ser^84^	Siderophore peptide	*K. pneumoniae*	Thomas et al., 2004
	MccM	7284^(d)^ 8115^(e)^	Linear trimer of N-2,3-(dihydroxybenzoyl)-L-serine (DHBS) anchored at the C-terminal Ser^77^	Siderophore peptide	*E. coli* Nissle 1917	Vassiliadis et al., 2010
	MccH47	4865^(d)^ 5696^(e)^	Linear trimer of *N*-2,3-(dihydroxybenzoyl)-L-serine (DHBS) anchored at the C-terminal Ser^60^	Siderophore peptide	*E. coli* Nissle 1917	Vassiliadis et al., 2010

*^(a)^Average masses with the cysteines involved in disulfide bonds when relevant.^(b)^Also termed thiazole-oxazole modified microcin (TOMM).^(c)^Putative structure.^(d)^Molecular mass without PTM.^(e)^Molecular mass including the DHBS trimer PTM.*

However, since there is a finite number of entry points and potential targets within a bacterium, microcins and antibiotics can share similar bacterial receptors and pathways to reach their intracellular targets. Moreover, as for antibiotics, the application of specific microcins might be curtailed by the development of resistance (Cotter et al., 2013). Thus, bacteria might evolve common mechanisms of resistance against microcins and antibiotics. This review will highlight the potential of microcins as an alternative to antibiotics to fight against bacterial resistance in Gram-negative pathogens and discuss the possibilities of cross-resistance and co-resistance occurrence in Gram-negative bacteria.

## Characteristics of Microcins

Bacteriocins that are produced by both Gram-positive and Gram-negative bacteria have been defined by James et al. (2013) as ribosomally synthesized peptides capable of mediating inhibitory effects against bacteria. In *Enterobacteriaceae* and more specifically in *E. coli*, microcins (for extensive reviews see Baquero and Moreno, 1984; Duquesne et al., 2007a; Baquero et al., 2019) have been shown to be produced along with colicins, which are large antibacterial proteins (Cascales et al., 2007). To distinguish them from colicins, the name “microcin” was coined since their first discovery (Asensio and Perez-Diaz, 1976), based on their smaller size of less than 10 kDa. Such as most bacteriocins, microcins are active against phylogenetically related bacteria including enteropathogenic *Klebsiella*, *Shigella*, *Salmonella* and *E. coli*, notorious for their capacity to develop antibiotic resistances, and considered serious and urgent threats by the CDC. These Gram-negative bacteriocins are ubiquitously distributed in Nature and their production is consistently observed in multiple genera. Those include *Escherichia*, *Salmonella*, *Shigella*, *Klebsiella*, *Enterobacter*, and *Citrobacter* (Gordon and O’Brien, 2006; Gordon et al., 2007; Budic et al., 2011; Drissi et al., 2015; Wang et al., 2016; Cheung-Lee et al., 2019). The development of DNA sequencing methods and the availability of an increasing number of genomes revealed that clusters of genes orthologous to microcin biosynthesis and self-immunity genes are widespread in bacteria. Indeed, analogs of historically described microcins produced by *Enterobacteriaceae*, essentially in the RiPP family, have been predicted and most often deeply characterized in other Gram-negative bacteria including human pathogens, *Helicobacter* (Bantysh et al., 2014), *Burkholderia* (Knappe et al., 2008), *Pseudomonas* (Metelev et al., 2013), *Klebsiella* (Metelev et al., 2017a, b; Travin et al., 2020), *Acinetobacter* (Metelev et al., 2017a), *Citrobacter* (Cheung-Lee et al., 2019), or in the symbiotic nitrogen-fixing bacterium *Rhizobium* (Travin et al., 2019) (Supplementary Figure S1A). They were even predicted in Gram-positive bacteria and cyanobacteria (Bantysh et al., 2014). This points that a sharp distinction between bacteriocins from Gram-positive and Gram-negative bacteria is artificial and that the chemical diversity of microcin-like peptides is intended to expand rapidly.

### The Two Classes of Microcins

Compared to the huge number of Gram-positive bacteriocins, microcins are distinguished by a high structural heterogeneity inside a restricted number of identified and well-characterized representatives. A widely accepted classification was proposed by Duquesne et al. (2007a) based on both the peptide size and degree of posttranslational modification (PTM). The known microcins are grouped in two classes, class I with molecular masses below 5 kDa and the presence of extensive PTM and class II with molecular masses between 5 and 10 kDa that can be modified or not (Table 1). A brief description of the microcins from the two classes is provided below to help following the next sections. For more detailed overview of the microcins, see two recent reviews (Baquero et al., 2019; Li and Rebuffat, 2020).

**FIGURE 1:**
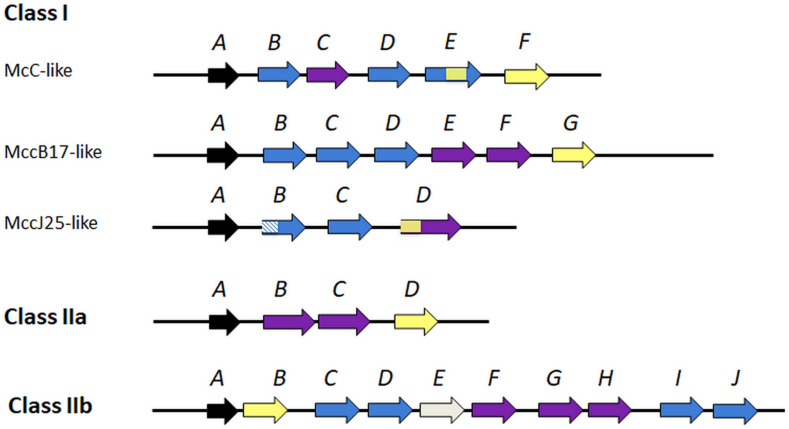
A schematic representation of archetypical organization of microcin and microcin-like gene clusters. Arrows indicate individual microcin genes; arrows are not drown to scale and their direction does not necessarily indicate the direction of transcription that can change between homologous specific gene clusters. The A genes code for the precursors. Genes coding for microcin PTM enzymes and for export systems (efflux pumps, ABC exporters) that expel the microcins out of the producers are in blue and in violet, respectively. Genes whose products contribute to self-immunity of the producing strains (either immunity proteins or exporters/efflux pumps) are colored yellow. When genes code for proteins ensuring simultaneously two functions, they harbor the two corresponding colors. The gene coding for RRE, which ensures leader peptide recognition in MccJ25 and MccJ25-like peptides is shown as hatched motif. The functions of the different PTM enzymes are indicated as follows, taking McC, MccB17, MccJ25 and MccE492 as models. McC and analogs: *mccB* product ensures MccA adenylation, *mccD-* and *mccE-*encoded enzymes (MccD and MccE N-terminal domain) are required for phosphate modification with propylamine; MccB17 and analogs: *mcbBCD-*encoded three-component synthetase catalyzes dehydration and cyclization to form azolines, which are subsequently oxidized to azoles; MccJ25 and analogs: *mcjC* product acts as a lasso cyclase that closes the macrolactam ring through an isopeptide bond and *mcjB* product is a leader peptidase; MccE492 and siderophore peptides: *mceCDIJ* are required for PTM with *mceC* encoding a glycosyltransferase that ensures glycosylation of enterobactin and *mceD* an enterobactin esterase that cleaves the glycosylated enterobactin macrolactone ring into its linear derivatives. *mceIJ* are involved in attachment of the PTM to MccE492 C-terminus. The function of *mceE* gene (gray) is undefined.

Class I assembles three plasmid-encoded microcins that have been well structurally characterized as RiPPs (Supplementary Figure S1A): microcin C (McC) a nucleotide peptide, microcin B17 (MccB17) a linear azol(in)e-containing peptide, and microcin J25 (MccJ25), a lasso peptide. McC is presently the only nucleotide member of the family. However, similar biosynthetic gene clusters are distributed within bacterial genomes (Bantysh et al., 2014), which suggests an unexplored diversity for such peptides. McC is produced by *E. coli* cells harboring the *mccABCDEF* gene cluster (Figure 1) under a *mccA*-encoded formylated heptapeptide precursor, which is further modified (Guijarro et al., 1995; Severinov and Nair, 2012) and processed into a structural mimic of aspartyl adenylate which is the toxic entity (Kazakov et al., 2008) (Supplementary Figure S1A). MccB17 is produced as a 69 amino acid precursor by *E. coli* strains bearing the *mcbABCDEFG* gene cluster (Figure 1). Mature MccB17 contains 43 amino acids that are structured into thiazole and oxazole heterocycles (4 thiazoles and 4 oxazoles rings either isolated or fused into oxazole/thiazole- and thiazole/oxazole-bis-heterocycles) by the PTM enzymes (Li et al., 1996; Ghilarov et al., 2019) (Supplementary Figure S1A). Such heterocycles are also found in hybrid non-ribosomal peptide-polyketide natural products such as the anti-tumor drug bleomycin, as well as in RiPPs such as cyanobactin (McIntosh and Schmidt, 2010) or streptolysin (Mitchell et al., 2009), forming the LAP [also termed thiazole/oxazole-modified microcin (TOMM)] peptide family (Melby et al., 2011). Microcin B-like bacteriocins produced by *Pseudomonas, Klebsiella* and *Rhizobium* have been reported (Metelev et al., 2013, 2017b; Travin et al., 2019). MccJ25 was isolated first from the *E. coli* strain AY25 isolated from an infant feces bearing the *mcjABCD* gene cluster (Salomón and Farias, 1992) (Figure 1). Its maturation from a 58 amino acid precursor into a 21 amino acid lasso peptide is ensured by two enzymes, McjB and McjC, encoded in the microcin gene cluster (Duquesne et al., 2007b; Yan et al., 2012). This unique lasso topology, which is characterized by threading of the C-terminal tail through a seven to nine lactam ring closed by an isopeptide bond, is locked in place with the two bulky side chains of Phe and Tyr aromatic amino acids for MccJ25 (Rosengren et al., 2003) (Supplementary Figure S1A). It is responsible for the sturdiness of MccJ25 and is required for its antibacterial activity (Rebuffat et al., 2004; Wang and Zhang, 2018). Genome mining approaches have revealed a wide distribution of lasso peptides in Gram-positive and Gram-negative bacteria (Maksimov et al., 2012; Hegemann et al., 2013; Tietz et al., 2017; Cheung-Lee and Link, 2019). Many lasso peptides produced by proteobacteria do not show antibacterial activity (Hegemann et al., 2013). This questions their ecological role or can be due to difficulty to decipher the reasons for their narrow activity spectrum.

Class II microcins form a more homogeneous group than their class I cousins (Table 1 and Supplementary Figures S1B,C), although they are subdivided into class IIa, encompassing MccL (Pons et al., 2004), MccN/24 (Kaur et al., 2016), MccPDI (Eberhart et al., 2012), MccS (Zschüttig et al., 2012) and MccV (Gratia, 1925), and class IIb (MccE492, MccH47, MccM, Vassiliadis et al., 2010). MccN was formely termed Mcc24 (O’Brien and Mahanty, 1994) and is termed MccN/24 in this review. What distinguishes class IIa from class IIb is the presence or not of a siderophore moiety derived from enterobactin anchored at the peptide C-terminal serine carboxylate (Supplementary Figures S1B,C). This catechol-type siderophore PTM sparked coining the name “siderophore microcins” to class IIb microcins (Rebuffat, 2012). Class II microcins result from a proteolytic processing of a precursor with a leader peptide extension, which occurs at a conserved double-glycine (or Gly-Ala) cleavage site, concomitantly with secretion. They have molecular masses between 5 and 10 kDa and exhibit high amino acid sequence similarities, even between class IIa and IIb (Supplementary Figures S1B,C). For examples, the class IIa unmodified MccV and MccN/24 possess high sequence similarities with the class IIb MccH47 and MccE492, respectively, although they do not carry a C-terminal PTM (O’Brien, 1996; Corsini et al., 2010). It was suggested that the conserved C-terminal sequence of these microcins can direct the presence or not of the siderophore PTM and that the C-terminal regions of MccV and MccH47 can be interchanged (Azpiroz and Laviña, 2007). It was further proposed that both class IIa and IIb microcins possess a modular structure (Azpiroz and Laviña, 2007; Morin et al., 2011).

Class IIa microcins have been characterized from *E. coli* strains from various origins. The MccN/24 producer is an uropathogenic *E. coli* (Kaur et al., 2016) and the MccL producer comes from poultry intestine (Sablé et al., 2003), while MccS is produced by a probiotic strain, *E. coli* G3/10 (Symbioflor2^®^; DSM17252) (Zschüttig et al., 2012). The producing strains are in some cases multi-microcin producers, such as *E. coli* LR05 that secretes MccB17, MccJ25 and the uncharacterized MccD93 in addition to MccL (Sablé et al., 2003). Their gene cluster organization includes the four basic genes only, one structural gene encoding the precursor peptide, two export genes and one immunity gene (Zschüttig et al., 2012) (Figure 1). If the five class IIa microcins are all devoid of PTMs, they are also all except MccN/24, stabilized by one (MccV) or two (MccL, MccPDI, MccS) disulfide bonds (Yang and Konisky, 1984; Sablé et al., 2003; Gerard et al., 2005; Morin et al., 2011; Zschüttig et al., 2012) (Table 1 and Supplementary Figure S1B).

Contrasting with class IIa and class I, class IIb microcins (Supplementary Figure S1C) are chromosome-encoded (Poey et al., 2006). MccE492 is secreted by *Klebsiella pneumoniae* human fecal strain RYC492 (de Lorenzo, 1984) bearing the *mceABCDEFGHIJ* gene cluster (Destoumieux-Garzón et al., 2006; Vassiliadis et al., 2007; Nolan and Walsh, 2008) (Figure 1). It is the first siderophore microcin to be characterized (Thomas et al., 2004), although it was primarily described as an unmodified peptide (Wilkens et al., 1997). Actually, it was shown further to be secreted under both modified and less active unmodified forms, due to its PTM process (Vassiliadis et al., 2007). The MccE492 PTM was identified as a glucosylated linear trimer of *N*-(2,3 dihydroxybenzoyl)-L-serine (DHBS) linked to the C-terminal serine carboxylate (Supplementary Figure S1C). The functions of the enzymes involved in establishment of the MccE492 PTM, MceC, MceD, MceI/MceJ, were identified (Vassiliadis et al., 2007; Nolan and Walsh, 2008). MccH47, initially isolated from the human fecal *E. coli* strain H47 (Laviña et al., 1990) and MccM were both characterized as siderophore microcins produced by several *E. coli* strains, including the probiotic strain Nissle, 1917 (Mutaflor^®^) (Vassiliadis et al., 2010). MccH47 and MccM carry the same PTM as MccE492 (Vassiliadis et al., 2010). Siderophore microcins possess a modular structure, where the N-terminal region is responsible for their cytotoxicity and the C-terminal region, which carries the siderophore moiety, is involved in recognition and uptake. For an overview on siderophore microcins, see Massip and Oswald (2020).

### Biosynthesis of Microcins

Microcin production takes place in the stationary phase (Baquero and Moreno, 1984) of bacterial growth, with the exceptions of MccE492 (de Lorenzo, 1984) and MccPDI (Eberhart et al., 2012). They are encoded by gene clusters, which exhibit a conserved organization, but contain a variable number of genes ranging from four to ten, according to the presence or not of PTMs on the mature microcin (Figure 1). These gene clusters are generally plasmid-borne, except the chromosomally encoded class IIb microcins. The general biosynthetic pathway of microcins (which also applies to other bacteriocins) starts with the ribosomal synthesis of a precursor peptide that is typically composed of two regions, an N-terminal leader part and a core region. The core peptide of modified microcins, which belong to the wide RiPP family, is the region where the PTMs take place (Montalbán-López et al., 2020). In some cases, such as the siderophore microcins, the modifications may result from the non-ribosomal pathway, making these microcins a rare bridge spanning ribosomal and non-ribosomal biosynthesis pathways (McIntosh et al., 2009). The leader is involved in binding to or activation of many of the PTM enzymes, but also maintains the maturing peptide inactive during the process (Arnison et al., 2013), thus contributing to the protection of producing cells as regard their own toxic microcin. For many modified microcins (MccJ25, McC), this binding involves a peptide binding domain (RiPP precursor peptide recognition element, RRE), also present in a wide proportion of RiPP PTM enzymes and similar to a small protein involved in the biosynthesis of the RiPP pyrroloquinoline quinone (PQQ) (Burkhart et al., 2015; Sikandar and Koehnke, 2019). Recently, the crystal structure of the McbBCD synthetase ensuring the extensive modifications in MccB17 was solved, deciphering the organization and functioning of such a multimeric heterocyclase-dehydrogenase catalytic complex at the molecular level and affording the spatial relationships between the two distinct enzymatic activities and the leader peptide binding site (Ghilarov et al., 2019).

In all but a few cases, and irrespective of if the microcin is modified or not, maturation requires removal of the leader region to give the active bacteriocin (Drider and Rebuffat, 2011). This proteolytic cleavage is performed either before and independently of (class I), or concomitantly with (class II) export of the mature microcin (Beis and Rebuffat, 2019). It can be ensured either (i)- concomitantly with the PTM establishment by one of the dedicated enzymes (MccJ25 leader is cleaved off by the McjB leader peptidase encoded in the microcin gene cluster (Yan et al., 2012), or (ii)- by a protease from the producer, which is not encoded in the microcin gene cluster (MccB17 leader is cleaved off before export by the conserved proteins TldD/TldE which assemble as a heterodimeric metalloprotease to ensure this function) (Ghilarov et al., 2017), or (iii)- by a bifunctional ATP binding cassette (ABC) transporter of the peptidase-containing ATP-binding transporters (PCAT) family, which is encoded in the microcin gene cluster (cleavage of the class II microcin leader peptides is performed simultaneously with export of the maturated microcins by an ABC exporter endowed with an N-terminal protease extension) (Håvarstein et al., 1995; Massip and Oswald, 2020).

### Self-Immunity of Microcin Producers

Microcin gene clusters vary in the number of genes contained and the presence of genes encoding PTM enzymes, and they all carry genes ensuring self-immunity (Figure 1). Each microcinogenic strain is protected against its arsenal of microcins and the self-immunity mechanisms differ from one microcin to another. For instance the self-immunity mechanism to McC is complex and relies on the products of three genes *mccC*, *mccE*, and *mccF* that ensure export of unprocessed microcin outside the cells (MccC pump) and modification of processed McC (MccE and MccF enzymes) (see section mechanisms of resistance) (Novikova et al., 2010; Agarwal et al., 2011, 2012). By contrast, the immunity mechanism to MccL depends on a single gene *mclI* that encodes an immunity protein (Sablé et al., 2003). Overall, self-immunity of the producers relies either on specific immunity proteins encoded in the gene clusters that bind to the toxic entities making them inefficient, or on efflux systems, mainly ABC transporters, which ensure export of the microcins to the external medium and simultaneously self-immunity of the producing bacteria. As examples, self-immunity to MccJ25 is provided exclusively by McjD, a highly specific ABC exporter which ensures simultaneously export of the microcin (Beis and Rebuffat, 2019), while full self-immunity to MccB17 requires both an immunity protein McbG and an ABC exporter McbEF (Collin and Maxwell, 2019).

## Mechanisms of Action

**FIGURE 2:**
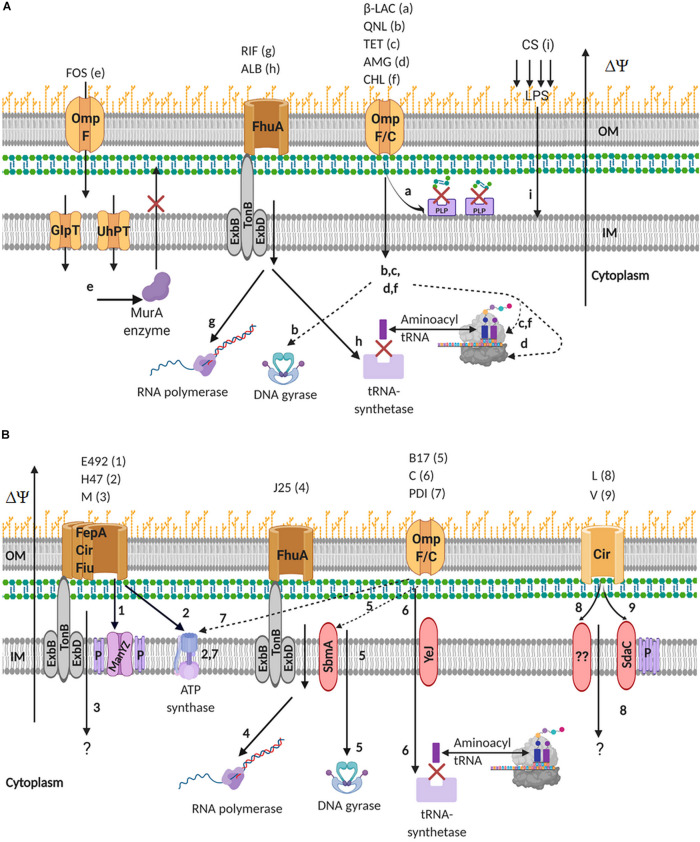
Mechanisms of action of antibiotics **(A)** and microcins **(B)** against Gram-negative bacteria showing the membrane proteins involved in uptake into sensitive bacteria and the final targets. β-LAC, β-lactams; QNL, quinolones; TET, tetracycline; AMG, aminoglycosides; FOS, fosfomycin; CHL, chloramphenicol; CS, colistin; RIF, rifampicin; ALB, albomycin; P, pore; LPS, lipopolysaccharide. A letter and a number are assigned to each antibiotic and each microcin respectively, which are used in the scheme to identify the path they follow for their killing activity.

Comparison of the mechanisms used by antibiotics and microcins to kill sensitive bacteria shows that they may share different bacterial receptors, translocators and final targets (Table 2 and Figure 2). Thus, it is obvious that these two groups of antimicrobials may cross in several mechanisms of action. However, it is also expected that several mechanisms of action of microcins are very specific and are not involved in the inhibition activity of antibiotics. This characteristic is particularly relevant to address in terms of the risk of cross-resistance between microcins and antibiotics. These similarities and differences are highlighted below.

**TABLE 2:** Comparison of the mechanisms involved in the antibacterial activity and the bacterial resistance for well characterized microcins and for conventional antibiotics sharing common targets with microcins.

Antibiotic/Microcin	Mechanisms of action		Mechanisms of resistance	
	Function impaired/*Target*	Uptake system (OM/IM)	Process/*Target*	Mechanism
Penicillins Cephalosporins Beta-lactams	– Bacterial cell wall disruption/*Peptidoglycan breaking*	– Porins; self-promoted pathway	– Inactivation/β*-lactam ring* – Mutations/*TonB Porins* – Efflux pumps overexpression	– β-lactam ring cleavage by β-lactamases – Decrease of uptake of the antibiotic due to modifications of TonB sequence – Decrease of uptake of the antibiotic – Pumping out of the antibiotic
Fosfomycin	– Bacterial cell wall/*Peptidoglycan biosynthesis: UDP-N-acetylglucosamine enolpyruvyl transferase, MurA* – Sugar transport into the cytoplasm	– GlpT, UhpT sugar transporters	– Mutations/*Mur A*	– Cys-Arg mutation in MurA active site – Mutations in GlpT, UhpT transporters
Polymixins Colistin/polymixin E	– Membrane permeabilization/*LPS binding leading to detergent effect* – Endotoxin neutralization	– Porins	– Enzymatic modification/*LPS* – Efflux pumps overexpression	– Modification of LPS by the MCR1 phosphoethanolamine transferase – Pumping out of the antibiotic
Rifamycins Rifampicin	– Protein synthesis- Transcription step/*β subunit of RNAP*	– Siderophore receptor FhuA – TonB system	Mutations./*RNAP β subunit*	– Mutations in *rpoB* gene
Streptolydigin	– Protein synthesis-Transcription step/*Inhibition of RNAP catalytic function by binding β and β′ subunits*	– Porins	– Mutations/*RNAP β and β′ subunits*	– Mutations in *rpoB* and *rpoC*
Albomycin	– Protein synthesis – Translation step/*Aminoacyl t-RNA synthetase*	– Siderophore receptor FhuA – TonB system	– Enzymatic modification/*Processed albomycin*	– Acetylation of processed albomycin by transacetylase RimL
Quinolones (nalidixic acid, ciprofloxacin, norfloxacin,…)	DNA replication/*Type II topoisomerases (DNA gyrase, topoisomerase IV*)		– Mutations – Protein interactions/*DNA gyrase, topoisomerase IV* – Enzymatic modification/*Piperazine ring* – Mutations/*Porins* – Efflux pumps overexpression	– Mutations in *gyrA, gyrB* or *parC, parE* (Ser^83^ in GyrA) – Protection of DNA gyrase and topoisomerase IV by the gyrase interacting protein qnr – Piperazine ring acetylation (AAC(6‘)-Ib-c) – Decrease of uptake of the antibiotic – Pumping out of the antibiotic
Chloramphenicol	– Protein synthesis/*Binding to 50S ribosome subunit inhibiting the formation of peptide bonds*	– Membrane transporter	– Enzymatic modification/*Chloramphenicol*	– Acetylation by chloramphenicol acetyltransferases CATs
Aminoglycosides	– Protein synthesis – Translation step/*Binding to 30S ribosome subunit*		– Enzymatic modification/*Aminoglycosides*	– Acetylation by acetyltransferases (AACs) – Phosphorylation by phosphotransferases (APHs) – Adenylation by nucleotidyltranferases (ANTs)
Tetracyclines	Protein synthesis -Translation step/*Binding to 30S ribosome subunit that blocks aminoacyl-tRNA binding to RNA-ribosome complex*	– Porins OmpF, OmpC	– Resistance genes acquisition – Enzymatic modification/*Tetracyclines* – Efflux pumps expression	– Acquisition of *tet*, or *otr* resistance genes leading to production of ribosomal production proteins Tet – Methylation by rRNA methylase – Pumping out of the antibiotic
MccJ25	– Protein synthesis-Transcription step/*Binding to β′ subunit of RNAP (secondary channel)*	– Siderophore receptor FhuA – TonB system – SbmA	– Mutations/*RNAP β′ subunit* – Efflux pumps expression	– Mutation in *rpoC* that encodes RNAP *β′* subunit (T^931^I) and additional mutations (Q^921^P, T^934^M, H^936^Y) – Pumping out of the microcin by ABC exporters (McjB, YojI)/TolC
MccB17	– DNA replication and topology maintenance/*Binding to DNA gyrase*	– Porin OmpF/ – SbmA	– Mutations/*GyrB, GyrA OmpF, OmpR SbmA* – Efflux pumps expression	– Mutations in GyrB and GyrA: GyrB (W^751^R): full resistance; GyrB (K^447^E), GyrA (S^83^W): partial resistance – Mutations in *omp*F and *omp*R – Pumping out of the antibiotic by ABC exporter (McbEF)
McC	– Protein synthesis – Translation step/*Aspartyl-tRNA synthetase (Asp-RS)*	– Porin OmpF/YejABEF	– Inactivation of the antibiotic *Processed McC* – Efflux pumps expression	– Acetylation of processed McC by transacetylases [either encoded in McC gene cluster (MccE) or chromosome-encoded (RimL)] – Cleavage of the heptapeptide-nucleotide amide bond by carboxypeptidases [serine carboxypeptidase encoded in McC gene cluster (MccF)] – Cleavage of the phosphoramide bond in aspartamide adenosine by histidine triad hydrolases – Pumping out of the antibiotic by ABC exporter MccC
MccE492	– Inner membrane bilayer permeability/*Formation of channels* – Sugar transport/*Binding to inner membrane components of mannose phosphotransferase system permease (ManPTS)*	– Siderophore receptors FepA-, Cir-, Fiu-TonB system	– Mutations in uptake system at the inner membrane/*Catechol siderophore receptors* – Mutations in mannose uptake system/*ManXYZ*	– Mutations/deletions in FepA, Cir, Fiu, TonB – Deletion of inner membrane complex ManYZ
MccH47	– Energy production (ATP synthesis)/*Binding to F_0_ subunits of ATP synthase*	– Siderophore receptors FepA Cir Fiu - TonB system	– Mutations in uptake system/*Catechol siderophore receptors*	– Mutations/deletions in FepA, Cir, Fiu
MccL	Membrane potential	– Siderophore receptor Cir -TonB system/SdaC	– Mutations in uptake systems/*Catechol siderophore receptor*	– Mutations in Cir, TonB
MccV	Inner membrane	– Siderophore receptor Cir -TonB system/ – SdaC	– Mutations in uptake systems/*Catechol siderophore receptor*, *SdaC*	– Mutations in Cir, TonB, SdaC
MccPDI	– Energy production/*ATP synthase* – Inner membrane permeability	– Porin OmpF	– Mutations/*Thiol-redox enzymes*, *OmpF*	– Mutations in *dsbA*, *dsbB* encoding thiol-redox enzymes making S-S bonds

### The Uptake Systems

The first obstacle to be overcome by an antimicrobial compound to reach its final target is the bacterial cell envelope (Collet et al., 2020). The extent of this barrier varies according to the target to be reached, the chemical structure of the antimicrobial compound and the bacterial species. For Gram-negative bacteria, antimicrobials have to pass first the outer membrane. Then, they can access the cytoplasmic membrane bilayer (inner membrane) and either insert inside or cross it for those antimicrobials having intracellular targets. Many antibiotics are hydrophilic compounds of low molecular mass and uptake across the outer membrane is ensured by passive diffusion using pores formed by specific β-barrel membrane proteins called porins. Porins are the most abundant proteins of the outer membrane in Gram-negative bacteria. They are classified as non-specific (general porins) and specific (selective porins), according to their threshold size and amino acids lining the aqueous channel (Choi and Lee, 2019). The transport varies according to the size, charge and hydrophilicity of the molecule. Recently, the dual function of the porin OmpF both as receptor and translocator for the pore-forming colicin N, has been elegantly demonstrated (Jansen et al., 2020). However, more hydrophobic or higher molecular mass compounds above the porin threshold require other strategies, among which hijacking receptors or transporters required for vital functions is a major one. Indeed, Gram-negative bacteriocins, colicins and microcins, widely parasitize such receptors to enter the periplasmic space, and particularly those involved in iron import. This receptor hijacking qualifies many microcins as “Trojan horse” compounds, as they mimic vital compounds that require being imported in cells, to penetrate sensitive bacteria (Duquesne et al., 2007a; Nolan and Walsh, 2008; Severinov and Nair, 2012).

Iron acquisition is an essential factor for microbial life. However, under aerobic conditions, free iron availability is limited by the very low solubility of ferric iron, and especially within a host, where iron is competed for by both the microbial community and the host (Wilson et al., 2016). To secure iron, bacteria have evolved to develop efficient Fe(III)-chelating agents (K_*a*_ ranging from 10^23^ to 10^52^), termed siderophores, to scavenge iron from their surrounding environment and import it. A study by Lewis et al. (2010) showed that siderophores are sufficient for allowing the culture of bacteria previously unculturable in laboratory conditions. Siderophores are non-ribosomally synthesized (Crosa and Walsh, 2002) and are important for enteropathogen survival (Hantke, 2003). Concomitantly, iron availability has been observed to regulate MccE492 gene expression (Marcoleta et al., 2013). The resulting Fe(III)-siderophore complex is then internalized by the producing strains via high affinity siderophore receptors anchored at the outer membrane, which are specifically involved in this function, but also ensure other strategic roles in microbial communities (Kramer et al., 2019). Siderophore receptors consist of a 22-stranded antiparallel β-barrel with external loops serving as ligand binding sites and an N-terminal globular domain forming a plug that occludes the barrel (Krewulak and Vogel, 2008). They are specific to the different siderophore chemical types, such as FhuA for ferrichrome or Cir, Fiu, and FepA for catechol siderophores in enterobacteria. These receptors are coupled to the TonB-ExbB-ExbD three-component machinery anchored at the inner membrane (TonB system), which transfers the energy source from the proton motive force of the cytoplasmic membrane to the outer membrane (Krewulak and Vogel, 2008), thus permitting active transport.

All microcins, whatever they are of class I or II, use either the siderophore receptor or the porin path to reach their final target (Figure 2B). Siderophore microcins uptake requires the FepA-, Cir-, Fiu-TonB systems, with FepA having the most important role (Destoumieux-Garzón et al., 2006; Azpiroz and Laviña, 2007; Vassiliadis et al., 2010). Unmodified microcins use the Cir-TonB system (MccV, MccL) (Chehade and Braun, 1988; Morin et al., 2011), or the porin OmpF which screens incoming products in a non-specific manner (Sato et al., 2000; Kaeriyama et al., 2006) (MccPDI) (Zhao et al., 2015), while class I microcins either use FhuA (MccJ25) (Pugsley et al., 1986; Salomón and Farías, 1993; Mathavan et al., 2014), or OmpF (MccB17, McC) (Laviña et al., 1986; Novikova et al., 2007) to reach the periplasmic space (Figure 2B). In the case of loss of function of the TonB system, MccE492, MccH47, and MccM retain antimicrobial activity, suggesting the involvement of another translocator, such as the TolA-TolQ-TolC system known to mediate the import of certain colicins (Lazdunski et al., 1998). Similar observations were made for MccL and MccV (Gerard et al., 2005; Morin et al., 2011), suggesting that the function of the ExbB protein could be replaced by its homolog TolQ in TonB-dependent microcin activity. However, although the presence of the siderophore PTM enhances its efficiency, the non-modified form of MccE492 (without the C-terminal siderophore) is also able to kill sensitive bacteria, but at a significantly lower level. On their side, antibiotics, which are essentially low molecular mass hydrophobic compounds, are most often transported inside target bacteria via porin or iron siderophore receptor pathways (Table 2).

### Mechanisms of Action Common to Antibiotics and Microcins

#### Disruption of the Cytoplasmic Membrane

Permeabilization and/or disruption of the bacterial cytoplasmic membrane of Gram-negative bacteria is the main mechanism of action of the non-ribosomal peptide antibiotics polymyxins B and E (Table 2 and Figure 2A), which share a high degree of structural similarity (Schindler and Teuber, 1975). Polymixin E (also called colistin) binds to the lipopolysaccharide (LPS) both in the bacterial outer membrane and in the cytoplasmic membrane and this interaction is essential for cytoplasmic membrane permeabilization, cell lysis and the bactericidal activity of this antibiotic (Sabnis et al., 2019). It should be noted that all polymyxins are inactive against Gram-positive bacteria, except few species such as *Streptococcus pyogenes* (Trimble et al., 2016).

Several class II microcins target the inner membrane, by perturbing either its integrity using different mechanisms of peptide membrane interaction, or the proteins which are embedded. This constitutes at least the primary part of their mechanism of action (Table 2 and Figure 2B). Indeed, the final killing trajectory of MccE492 appears to stop at the inner membrane. MccE492 induces a rapid depolarization and permeabilization of *E. coli* cytoplasmic membrane, without provoking cell lysis (Lagos et al., 1993; Destoumieux-Garzón et al., 2006). It forms well-defined ion channels in planar phospholipid bilayers that are constituted of supramolecular peptide assemblies (Lagos et al., 1993; Destoumieux-Garzón et al., 2006). It also interacts with the mannose phosphotransferase system permease ManXYZ (Bieler et al., 2010), associating specifically with its inner membrane components ManYZ. Therefore, MccE492 both perturbs the inner membrane permeability and interferes with the transport of mannose to kill sensitive congeners. Besides, MccE492 is known to form amyloid fibrils (Bieler et al., 2005; Arranz et al., 2012; Aguilera et al., 2016) that play a role in modulating its antimicrobial activity. These aggregates have been observed more significantly with the unmodified form of MccE492, suggesting their formation is not only an additional mechanism of protection of the producer strain, but also may act as a toxin reservoir. MccV destabilizes the membrane potential (Yang and Konisky, 1984) and further interacts with an inner membrane transporter, the serine permease SdaC (Gerard et al., 2005), which is involved in serine transport and acts as a specific receptor for MccV. It can be suggested that a perturbation of serine transport in sensitive bacteria could result, or that SdaC could drive MccV to form channels in the inner membrane. MccE492 and MccV thus illustrate the combined use of two different mechanisms involving the inner membrane or its components to kill sensitive bacteria. MccL primary target is also the cytoplasmic membrane. It provokes disruption of membrane potential of *E. coli* cells, but without inducing permeabilization of the inner membrane (Morin et al., 2011). A potential inner membrane target for MccL has not been identified. Finally, it has to be mentioned that at higher concentrations than the MIC, MccJ25 induces perturbations of the cytoplasmic membrane permeability and disruption of the cytoplasmic membrane gradient in *Salmonella enterica* (Rintoul et al., 2001; Ben Said et al., 2020), and perturbation of the respiratory chain enzymes in *E. coli*, accompanied with stimulation of the production of reactive oxygen species (Bellomio et al., 2007).

#### Inhibition of Protein Biosynthesis

The bacterial 70S ribosome is composed of two ribonucleoprotein subunits forming the 30S and 50S subunits (Yoneyama and Katsumata, 2006). Aminoglycosides (AGs), such as streptomycin or gentamicin, and tetracyclines bind to the 16S ribosomal RNA of the 30S subunit (Chopra and Roberts, 2001; Krause et al., 2016). AGs bind to the A-site of the ribosome, causing inhibition of translation of mRNA by codon misreading on delivery of the aminoacyl-tRNA (Table 2 and Figure 2A). For their part, tetracyclines prevent incoming aminoacyl-tRNA from binding to the A site of the mRNA translation complex. As well, chloramphenicol inhibits protein synthesis by preventing the binding of t-RNAs to the A site of the ribosome (Kapoor et al., 2017). The bacterial ribosome is also the target for other antibiotic classes, such as the macrolides and ketolides or the streptogramins.

Contrasting with MccB17 and its *Pseudomonas* congeners which exert their antimicrobial activity by perturbing DNA topology setting up (see section below Inhibition of Nucleic Acid Biosynthesis), other MccB17-like bacteriocins perturb protein synthesis. Klebsazolicin from *K. pneumoniae*, which exhibits moderate antimicrobial activity against certain *E. coli*, *Klebsiella* and *Yersinia* strains (Metelev et al., 2013) targets the 70S ribosome and interferes with translation elongation. Moreover, it binds to the peptide exit tunnel, overlapping with the binding sites of macrolides or streptogramin-B. Similar to klebsazolicin, the MccB17-like phazolicin produced by *Rhizobium* sp., which exhibits narrow-spectrum antibacterial activity against some symbiotic bacteria of leguminous plants (Travin et al., 2019), also targets the 70S ribosome by obstructing the peptide exit tunnel, but through different binding mechanisms.

Albomycin, which consists of an antibiotic part linked to a siderophore moiety, inhibits aminoacyl t-RNA synthetases (aaRSs) that are essential for protein synthesis (Severinov and Nair, 2012) (Table 2 and Figure 2A). Similar, McC targets the aspartyl-tRNA synthetase (Metlitskaya et al., 2006), making it a translation inhibitor (Table 2 and Figure 2B). After having crossed the outer membrane thanks to the porin OmpF, McC requires the inner membrane ABC transporter YejABEF (Novikova et al., 2007) for its translocation within the cytoplasm. A comprehensive analysis by Vondenhoff et al. (2011) has shown that to mediate binding and translocation of substrates, the YejABEF transporter requires an N-terminal formyl-methionine and an arginine. These requirements are achieved with the formylated f-MRTGNAD heptapeptide part of the McC precursor. However, unlike other microcins, which are fully processed within the producing cells before export, further McC maturation is necessary within the target bacteria to attain its cytotoxic form. McC undergoes a double-step processing. First of which is the deformylation of the formylated heptapeptide precursor, essentially nullifying the detoxification process of its immunity protein *mccE*. This deformylation allows the second maturation step, which is ensured by broadly specific endoproteases PepA, PepB, and PepN, which remove the peptide moiety of the microcin. This last processing step releases the toxic entity, which is a non-hydrolyzable analog of aspartyl-adenylate (Asp-RS) that blocks aspartyl-tRNA synthetase and thus transcription (Kazakov et al., 2008). This subtle cheating mechanism nicely exemplifies the Trojan horse strategy used by microcins. Moreover, Ran et al. (2017) observed that when increasing the concentration until the mM level, McC was able to inhibit the activity of β-galactosidase, respiration chain dehydrogenases, and 6-phosphogluconate dehydrogenase without damaging the inner membrane, showing that McC develops a second mechanism of action that operates at higher concentrations.

#### Inhibition of Nucleic Acid Biosynthesis

Quinolone antibiotics (nalidixic acid, ciprofloxacin, …) inhibit DNA synthesis by targeting two essential type II topoisomerases, DNA gyrase and topoisomerase IV, and converting them into toxic enzymes that fragment the bacterial chromosome (Table 2 and Figure 2A). These interactions result in erroneous unwinding of DNA, introduction of double strand breaks and cell death (Fabrega et al., 2009). Besides, rifampicin inhibits DNA-dependent RNA polymerase (RNAP) activity by forming a stable complex with the enzyme. It binds in a pocket of the RNAP β subunit, deep within the DNA/RNA channel, while away from the active site. The inhibitor directly blocks the path of the elongating RNA when the transcript becomes two to three nucleotides in length. It thus suppresses the initiation of RNA synthesis (Campbell et al., 2001).

The target of MccB17 is also a topoisomerase (Table 2 and Figure 2B). MccB17 enters sensitive bacteria using the OmpF porin, diffuses through the periplasmic space and binds to the inner membrane transporter SbmA to be delivered into the cytoplasm (Laviña et al., 1986). It induces gyrase-dependent formation of a stable cleavage complex instead of the transient break that normally happens during the catalytic cycle. It causes covalent links between DNA gyrase and double stranded DNA, hence blocking DNA replication and maintenance. Similar to fluoroquinolones, MccB17 targets the cleavage of both DNA strands, which is a critical step in the DNA gyrase supercoiling cycle, but the MccB17-induced cleavage pattern is different from that of quinolones (for a review on MccB17 activity see Collin and Maxwell, 2019). The stringent role of the heterocycles in MccB17 activity has been evidenced (Roy et al., 1999). Introduction of an extra oxazole ring at position Ser^52^ in MccB17 results in 40% higher antibacterial activity than that of wild-type MccB17 (Roy et al., 1999). Bis-heterocycles play a particularly essential role, with the central MccB17 region that contains two thiazoles and a thiazole/oxazole forming the critical core for DNA cleavage (Collin and Maxwell, 2019). Moreover, the C-terminal part of MccB17 is crucial for both uptake by sensitive cells and DNA gyrase inhibition, while the N-terminal region is only moderately important for uptake (Shkundina et al., 2014). Interestingly, MccB17 congeners that belong to the LAP family of RiPPs do not share all similar mechanisms, targeting either DNA gyrase or the 70S ribosome. Indeed, MccB17-like compounds from *P. syringae* are active against *E. coli* and essentially *Pseudomonas* species including *P. aeruginosa*, through DNA gyrase inhibition (Metelev et al., 2013), while the other analogs do not (see section above “Inhibition of Protein Biosynthesis”).

Such as rifampicin, the lasso peptide MccJ25 targets the RNAP (Table 2 and Figure 2B). To reach its intracellular target, MccJ25 hijacks the ferrichrome receptor FhuA to cross the outer membrane (Mathavan et al., 2014) and is internalized into the cytoplam by the inner membrane protein SbmA. Finally, MccJ25 binds to the RNAP secondary channel, which connects the enzyme surface with the RNAP catalytic center, and through which nucleotide triphosphate substrates (NTP) migrate to the catalytic center (Adelman et al., 2004; Mukhopadhyay et al., 2004), whereby inactivating transcription in a partial competitive manner. The loop is involved in recognition and uptake of MccJ25 by the iron-siderophore transporter FhuA, while the macrolactam ring and C-terminal tail are responsible for binding to the RNA polymerase target (Destoumieux-Garzón et al., 2005; Semenova et al., 2005). The crystal structure of MccJ25 bound to *E. coli* RNAP was determined and the residues critical for the interaction were identified (Braffman et al., 2019). MccJ25 binds deep within the secondary channel, such as to clash with NTP binding and explaining the partial competitive mechanism of inhibition with respect to NTPs previously proposed (Mukhopadhyay et al., 2004). Besides, it was shown that at higher concentrations, MccJ25 induces perturbations of the cytoplasmic membrane permeability and disruption of the cytoplasmic membrane gradient of *S. enterica* Newport (Rintoul et al., 2001). At much higher concentrations, it can also stimulate the production of reactive oxygen species (Bellomio et al., 2007). This shows once again the multiple mechanisms brought into play by a given microcin, which both explains their high efficiency and suggests lower risks of resistance acquisition. Several antibacterial lasso peptides, have been shown to also target RNAP through binding to the secondary channel, although their different antibacterial activity spectrum. This is the case for capistruin produced by *Burkholderia thailandensis* and active against *Burkholderia* and *Pseudomonas* species (Knappe et al., 2008; Kuznedelov et al., 2011; Braffman et al., 2019), ubonodin from *B. ubonensis* and active against pathogenic members of the *B. cepacia* complex (Cheung-Lee et al., 2020), citrocin from *Citrobacter* sp., active against *E. coli* and *Citrobacter* sp. (Cheung-Lee et al., 2019). By contrast, acinetodin and klebsidin from human-associated strains of *Acinetobacter* and *Klebsiella*, display no activity or low activity against *K. pneumoniae*, while they bind RNAP (Metelev et al., 2017b), showing that the spectrum of activity of lasso peptide microcins appears to be driven by the uptake in target bacteria rather than the intracellular target. This is in agreement with the spectrum of activity of MccJ25 against a collection of *Salmonella* strains, which is associated mainly with differences in the FhuA sequences (Ben Said et al., 2020).

### Mechanisms of Action Specific to Microcins

MccH47 is bactericidal and targets the membrane bound F_0_ proton channel subunits of ATP synthase (Trujillo et al., 2001; Rodriguez and Laviña, 2003; Palmer et al., 2020), causing an unregulated influx of protons. It uses FepA-, Cir-, Fiu-TonB dependent receptors to reach its inner membrane target (Patzer et al., 2003). The mechanism of action of the class IIa MccPDI is poorly identified. It was told to require close bacterial proximity to be cytotoxic, hence the name PDI (Proximity Dependent Inhibition) (Eberhart et al., 2012), since co-cultures of producing and sensitive strains separated by a semi-permeable film inhibit its activity. Why proximity is required for activity is unknown, but it could be only a consequence of a concentration-dependence effect (Lu et al., 2019). MccPDI that uses the porin OmpF to cross the outer membrane (Zhao et al., 2015; Lu et al., 2019) was shown (Zhao et al., 2015) to require a functional ATP synthase for exerting its cytotoxic activity, while (Lu et al., 2019) proposed it would induce membrane damage.

### Mechanisms of Action Specific to Antibiotics

#### Inhibition of Cell Wall Formation

The cell envelope of Gram-negative bacteria consists of a phospholipid bilayer inner membrane that wraps the cytoplasm, and an asymetric outer membrane essentially composed of phospholipids at the inner leaflet and LPS at the outer leaflet, which protects the cell from the environment. In between is the periplasm that shelters a thin peptidoglycan layer (Collet et al., 2020). This double-membrane complex system and in particular the peptidoglycan, often called the cell wall, is a main target for antibiotics and antimicrobials. β-lactam antibiotics, which include in particular penicillins, cephalosporins and carbapenems, harbor the β-lactam ring in their structure that mimics the D-alanyl D-alanine terminal amino acid residues of the precursor subunits of the peptidoglycan layer, and so far interacts with penicillin binding proteins (PBPs). This induces a disruption of the peptidoglycan layer leading to the lysis of the bacterium (Kapoor et al., 2017). Besides, fosfomycin inhibits bacterial cell wall biosynthesis in an early stage; it integrates the cell and inactivates an essential enzyme in peptidoglycan synthesis (Dijkmans et al., 2017). β-lactams, mainly carpabenems and second, third and fourth generation of cephalosporins as well as fosfomycin have a broad spectrum antibacterial activity.

#### Inhibition of Folic Acid Metabolism

Trimethoprim and sulfonamides act at distinct steps in folic acid metabolism. Sulfonamides inhibit dihydropteroate synthase, which acts at an early step in folic acid biosynthesis in a competitive manner with higher affinity for the enzyme than the natural substrate, *p*-amino benzoic acid (PABA). For its part, trimethroprim inhibits dihydrofolate reductase, thus operating at a later stage of folic acid synthesis (Yoneyama and Katsumata, 2006).

## Mechanisms of Resistance and Potential Cross- and Co-Resistance Between Antibiotics and Microcins

**FIGURE 3:**
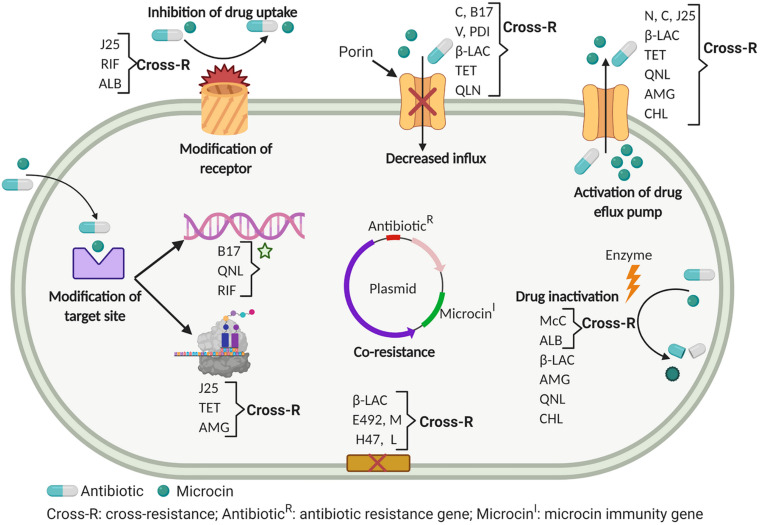
Mechanisms of cross-resistance and co-resistance of antibiotics and microcins in Gram-negative bacteria. β-LAC, β-lactams; QNL, quinolones; TET, tetracycline; AMG, aminoglycosides; FOS, fosfomycin; CHL, chloramphenicol; CS, colistin; RIF, rifampicin; ALB, albomycin.

Various mechanisms of resistance to antibiotics and/or to microcins are reported including essentially modifications of the cellular target by mutations or protein interactions, changes in the structure of the antimicrobial molecule, perturbations of binding or penetration of the antibiotic into sensitive cells and specific cell wall modifications. Several mechanisms are specific, but bacteria may use common mechanisms of resistance against microcins and antibiotics that could induce cross-resistance, which occurs when a single mechanism provides resistance to several antimicrobial molecules differing in their structures, simultaneously. In contrast, co-resistance occurs when two or more different resistance genes encoding several unrelated resistance mechanisms are located on the same genetic element (plasmid, transposon) (Chapman, 2003). In the following section, we describe different mechanisms of resistance and the possible occurrence of cross- and co-resistance between antibiotics and microcins (Table 2 and Figure 3).

### Prevention of Intracellular Accumulation of the Toxic Entity: Efflux Pumps and Decreased Uptake

On one side, outer membrane porins and inner membrane transporters, which are involved in the uptake of antibiotics and microcins into sensitive cells, and on the other side efflux pumps, which pump the toxic compounds out of the bacteria, both constitute a first line resistance strategy (Ghai and Ghai, 2018). Porins, which ensure passive uptake of substrates across the outer membrane (see section mechanisms of action above), serve as the first gate for many antibiotics and several class I and II microcins. Furthermore, efflux pumps can be specific for a single substrate or can confer resistance to multiple antimicrobials by facilitating their extrusion before they can reach their intended targets (Anes et al., 2015). In Gram-negative bacteria, overexpression of efflux pumps is one of the mechanisms of resistance to β-lactams (Amaral et al., 2014) and to quinolones encoded by *qep*A and *oqx*AB genes (Fabrega et al., 2009). Likewise, reduced porin levels, which induce decrease of antibiotic concentration inside sensitive cells, is another mechanism of resistance to β-lactams in Gram-negative bacteria (Pfeifer et al., 2010), including *K. pneumoniae* (Jacoby et al., 2004) and *P. aeruginosa* (Li et al., 1994). Besides, mutations and deletions of genes encoding porins induce resistance to antibiotics. Indeed, *ompF* mutant was resistant to several β-lactam antibiotics in some Gram-negative pathogens, including *E. coli* and the deletion of OmpA resulted in increased susceptibility to several antibiotics including β-lactams in *A. baumannii* (Smani et al., 2014).

For microcins, the *E. coli* ABC exporter of unknown function Yojl, mediates resistance to MccJ25 by pumping the microcin out of the cells with the help of TolC, maintaining its concentration below the toxic concentration (Delgado et al., 2005). Yojl is located at the inner membrane and is coupled to the TolC protein at the outer membrane which ensures the last export step, similar to the MccJ25 gene cluster-encoded ABC exporter McjD, which warrants both microcin export and self-immunity for the producing cells (Bountra et al., 2017; Beis and Rebuffat, 2019). Similarly, McC is expelled from producing cells through a major facilitator superfamily (MFS) efflux pump (Severinov and Nair, 2012). Thus, the activation of several efflux pumps simultaneously could induce a co-resistance to antibiotics and microcins.

The iron-siderophore receptor FhuA is not only required for iron import, but it is also a target for bacteriocins (colicin M, MccJ25) and antibiotics (albomycin, rifamycin). Indeed, FhuA external loops L3, L4, L7, L8, and L11 are involved in the sensitivity to colicin M and the antibiotics albomycin and rifamycin. So far, a further mutation, insertion or deletion in the sequence encoding these loops may induce a cross-resistance between colicin M and these two antibiotics (Wang et al., 2018). Concomitantly, MccJ25 was also shown to require a primary interaction with the FhuA external loops L5, L7, L8 and L11 for its recognition and further internalization via this receptor (Destoumieux-Garzón et al., 2005). The level of sensitivity to MccJ25 also varies depending on the acquisition of specific FhuA, with a maximal sensitivity obtained with *E. coli* FhuA, while several *Salmonella* serovars are resistant due to a lack of efficiency of their FhuA receptor for MccJ25 uptake (Vincent et al., 2004; Ben Said et al., 2020). Similarly, various mutations in FhuA, especially in the cork domain, were reported to reduce the uptake and consequently the sensitivity to albomycin (Endriss et al., 2003). It could thus be hypothesized too that cross-resistance can occur between MccJ25 and albomycin. Besides, membrane permeabilization induced by a synthetic cationic peptide (KFF)_3_K was shown to induce the sensitivity of MccJ25 resistant clinical isolates, thus making the microcin entry independent of FhuA and SbmA proteins (Pomares et al., 2010), and thus confirming that microcin uptake is the first source of resistance to MccJ25. Therefore, both uptake decrease of the toxic entity and pumping it out of the sensitive cells are efficient mechanisms to confer resistance to MccJ25.

Resistance to siderophore microcins which carry a catechol siderophore PTM is also primarily induced by uptake impairment (Thomas et al., 2004; Massip and Oswald, 2020). As seen before, MccE492, MccM and MccH47 are recognized and internalized in sensitive bacteria via the TonB-dependent FepA, Fiu and Cir iron-catecholate receptors. According to Thomas et al. (2004), a *fepA*, *fiu* double mutation, the triple *cir*, *fiu*, *fepA* mutation and the *tonB* mutation induce complete resistance to MccE492, MccM, and MccH47, while deletion of *exbB* and *exbD* does not affect the sensitivity to all three siderophore microcins (Vassiliadis et al., 2010). Although it does not carry a siderophore PTM, MccL requires the TonB dependent catecholate receptor Cir for uptake. Mutations/deletions in Cir and TonB, or suppression of the proton motive force, which is required for the TonB function, afford MccL resistance in *E. coli* and *Salmonella*, while the proteins involved in serine or sugar transport are not involved (Morin et al., 2011). On the other hand, a mutation in the energy transducer TonB was shown to reduce uptake and confer resistance to ceftazidime. Moreover, ceftazidime-resistant TonB mutants were shown to be cross-resistant to fluoroquinolones and lactivicin, a siderophore-conjugated non-β-lactam antibiotic (Calvopina et al., 2020). Thus, a high probability exists for a possible cross-resistance between these antibiotics and microcins.

Resistance to MccN/24 is afforded by mutations in genes encoding the outer membrane porin OmpF (Jeanteur et al., 1994), or the inner membrane transporter SdaC involved in serine uptake and used for MccV activity (Gerard et al., 2005). Resistance to MccPDI also involves OmpF and more precisely the K^47^G^48^N^49^ amino acid motif found in the predicted outer loop L1 of the porin (Zhao et al., 2015; Lu et al., 2019). In addition, mutations in DsbA and DsbB proteins, presumably involved in the formation of disulfide bonds in OmpF, induce resistance to MccPDI (Zhao et al., 2015). Mutations in *ompF* and *ompR* genes encoding OmpF induce a reduced sensitivity to MccB17. Moreover, a mutation in the *sbm*A gene encoding the inner membrane transporter SbmA, which translocates MccB17 from the inner membrane to the cytoplasm, induces high resistance to MccB17 (Laviña et al., 1986).

As regard the efflux systems involved in resistance to microcins, resistance to MccN/24 is controlled by the multiple antibiotic resistance (*mar*) operon (Carlson et al., 2001), which modulates efflux pump and porin expression via two encoded transcription factors, MarR and MarA (Sharma et al., 2017). MarA plays an important role in antibiotic resistance by activating the expression of the *acrAB-tolC* encoded efflux pump (Zhang et al., 2008) and also regulates biofilm formation (Kettles et al., 2019). Resistance to MccN/24 in *Salmonella* cells appears concomitantly with a multiple antibiotic resistance phenotype to ciprofloxacin, tetracycline, chloramphenicol and rifampicin (Carlson et al., 2001). So far, cross-resistance between MccN/24 and antibiotics raised above is quite possible.

Additional mechanisms involve specific cell wall modifications. Those include surexpression of capsule polysaccharides that can increase resistance to various antimicrobials including both antibiotics, in particular polymixins, and antimicrobial peptides (Campos et al., 2004). Interestingly, capsule polysaccharides are not involved in MccJ25 resistance of the YojI deficient strain (Delgado et al., 2005). Alterations of the LPS resulting in truncated LPS structures promote, among other pleiotropic effects, resistance to antimicrobial peptides and hydrophobic antibiotics (Pagnout et al., 2019).

### Changes in Target Sites

To allow DNA supercoiling, bacteria use two type II topoisomerases, DNA gyrase and topoisomerase IV, which are both the targets of quinolones. They form a ternary cleavage complex gyrase/DNA/quinolone, thus blocking DNA replication. Mutations in genes encoding DNA gyrase (*gyrA*, *gyrB*) and topoisomerase IV (*parC*, *parE*) lead to quinolone resistance. Besides, a plasmid-mediated protection of DNA gyrase and topoisomerase IV from the action of quinolones is ensured in a non-specific manner by the gyrase interacting protein Qnr. Qnr is a 218 amino acid pentapeptide repeat protein (PRP) encoded by *qnr* genes, which blocks the action of quinolones on the DNA gyrase and topoisomerase IV in a lesser extent (Fabrega et al., 2009; Jacoby et al., 2015). Indeed, one of these mutations is the well-known GyrB W^751^R mutation which induces resistance to quinolones and is also linked to resistance to MccB17 (Vizán et al., 1991). GyrB Trp^751^ is strongly implicated in the interaction of DNA gyrase with MccB17 (Heddle et al., 2001) and *gyrB* point mutation changing Trp^751^ for Arg leads to a protein variant resistant to MccB17 (del Castillo et al., 2001). Additionally, partial resistance to MccB17 is provided by mutations at position 83 in GyrA or 447 in GyrB (Jacoby et al., 2015). Consequently, cross-resistance to MccB17 and quinolones could occur. Otherwise, it is well known that immunity genes are responsible for protecting the producing bacteria from their own bacteriocin. Indeed, three genes *mcbE, mcbF*, and *mcbG* are involved in cell protection from endogenous and exogenous MccB17. Interestingly strains harboring these genes are shown to be highly resistant to fluoroquinolones (Tran and Jacoby, 2002). These mechanisms seem to be responsible for co-resistance to MccB17 and quinolones.

Mutation of the gene *rpo*B encoding the β′ subunit of RNAP (see section mechanisms of action above) induces resistance to rifampicin (Campbell et al., 2001; Goldstein, 2014). Likewise, alterations in the 30S or 50S subunit of the ribosome lead to resistance to antibiotics that act on these proteins, mainly tetracycline, chloramphenicol, streptolydigin and aminoglycosides (Kapoor et al., 2017). Similarly, first studies performed to understand the mechanism of action of MccJ25 have shown that a point mutation causing a substitution of Thr^931^ for Ile in the conserved segment of the *rpo*C gene coding for the largest RNAP subunit β′ conferred resistance to MccJ25, suggesting a mechanism involving occlusion of the RNAP secondary channel (Delgado et al., 2001; Yuzenkova et al., 2002). It was shown further from the crystal structure of the MccJ25-RNAP complex that MccJ25 binds within the RNAP secondary channel and interferes with the traffic of NTPs to the catalytic center (Braffman et al., 2019). Furthermore, additional *rpoC* mutations affecting amino acids in the conserved segments G, G′ and F and exposed into the RNAP secondary channel, also led to MccJ25 resistance *in vivo* and *in vitro*. While MccJ25 acts on the β′ subunit, and rifampicin on the β subunit, streptolydigin acts on both subunits. So far, a cross-resistance between MccJ25 and the above cited antibiotics mainly streptolydigin and rifampicin appears to be highly expected (Yang and Price, 1995; Temiakov et al., 2005).

For other antibiotics and microcins, no specific cross-resistance appears to be predictable. Chromosomally mediated colistin resistance occurs mainly via the addition of cationic moieties onto the negatively charged lipid A, while the plasmid mediated colistin resistance (MCR) is acquired via a plasmid-borne copy of an *mcr* gene. MCR-1 is the most prevalent MCR enzyme reported for the first time in 2015 followed by nine homologs described to date (Carroll et al., 2019). MCR-1-mediated colistin resistance confers protection against this last resort antibiotic via the presence of modified LPS within the cytoplasmic membrane, rather than the outer membrane (Sabnis et al., 2019). More precisely, the phosphoethanolamine transferase activity of MCR-1 adds a cationic phosphoethanolamine moiety to the anionic lipid domain A of LPS, which results in a net negative charge decrease and thus a lower affinity for the polymyxins.

Fosfomycin inhibits the bacterial cell wall synthesis at the early initiating step of the peptidoglycan synthesis. More specifically, it inhibits UDP-*N*-acetylglucosamine enolpyruvyl transferase (or MurA), the enzyme involved in transfer of the enolpyruvyl part of phosphoenolpyruvate to the 3′-hydroxyl group of UDP-*N*-acetylglucosamine, which is the first step in the biosynthesis pathway of peptidoglycan. Mutations in the *murA* gene confer resistance to fosfomycin due to the replacement of cysteine with aspartate in the active site of MurA, which prevents fosfomycin binding (Falagas et al., 2019). Moreover, resistance to fosfomycin can occur from chromosomal mutations in the structural genes that encode the GlpT and UhpT membrane transporters. GlpT and UhpT transport glycerol-3-phosphate and glycerol-6-phosphate sugars in bacteria, respectively and are used by fosfomycin to facilitate its entry in bacteria. These mutations block fosfomycin cell penetration (Falagas et al., 2019).

On the microcin side, the F_1_F_0_-ATP synthase has been shown to be the target of MccH47 (Rodriguez and Laviña, 2003) and MccPDI (Zhao et al., 2015). *E. coli* ATP synthase consists of a membrane-bound F_0_ sector, which ensures proton translocation, connected to a cytoplasmic F_1_ sector. They form a complex made up of eight different subunits, which are encoded by the *atp* operon, *atpIBEFHAGDC*. Three subunits form the F_0_ proton channel and five subunits the catalytic F_1_ domain. Mutations on genes *atpB*, *atpE*, *atpF* encoding the three subunits F_0_a, F_0_c, F_0_b respectively, which constitute the F_0_ proton channel, result in resistance to MccH47 (Rodriguez and Laviña, 2003). Furthermore, deletion of genes encoding subunits in the F_1_ and F_0_ domains of ATP synthase (*atpA* and *atpF* encoding F_1_α and F_0_b subunits, or *atpE* and *atpH* encoding F_0_c and F_1_δ subunits), result in a loss of susceptibility to MccPDI simultaneously to the loss of ATP synthase function (Zhao et al., 2015). None of these mechanisms appears to be shared between antibiotics and microcins.

### Inactivation of the Toxic Entity

Several Gram-negative bacteria produce different enzymes that are able to modify antibiotics and thus induce resistance, such as the very well-known β-lactamases, which disrupt the specific structure of β-lactams (Sawa et al., 2020). β-lactamases are classified into four classes including group 1 (class C) cephalosporinases, group 2 (classes A and D) broad-spectrum, inhibitor-resistant, and extended-spectrum β-lactamases as well as serine carbapenemases, and group 3 (class B) metallo-β-lactamases (Bush and Jacoby, 2010). Other enzymes including aminoglycosides modifying enzymes, such as phosphotransferases (APHs), nucleotidyltranferases (ANTs) and acetyltransferases (AACs), which phosphorylate, adenylate and acetylate these compounds, respectively could also be involved in development of resistance (Ramirez and Tolmasky, 2010).

Acetylation is a widespread and efficient mechanism of resistance against different antibiotics. Modification of the piperazine ring of the fluoroquinolones is induced by an acetylase AAC(6′)-Ib-cr, which provides one of the mechanisms of resistance of bacteria to quinolones (Fabrega et al., 2009). Chloramphenicol is also inactivated by acetylation which is performed by chloramphenicol acetyltransferases (CATs) (Smale, 2010). Acetylation is also a major mechanism of resistance to McC, then suggesting a high risk of cross-resistance between chloramphenicol and McC. Before its ultimate processing by non-specific aminopeptidases, which happens in sensitive cells to release the toxic non-hydrolyzable analog of aspartyl-adenylate, McC is exported outside the producer by the MccC pump and uptaken by sensitive cells using the porin OmpF and the inner membrane transporter YejABEF (see section Mechanisms of action). However, although most of produced McC is efficiently exported, intracellular processing also occurs inside the producing cells that ineluctably leads to the accumulation of the toxic entity that cannot be exported by the MccC pump and results in self-poisoning. Therefore, *E. coli mcc* gene clusters include genes (*mccE* and *mccF*) that encode proteins ensuring the self-immunity of the producer. The MccE acetyltransferase acetylates the α-amino group of processed McC, making it unable to bind to AspRS (Agarwal et al., 2011). So far, MccE makes *E. coli* simultaneously resistant to albomycin and McC (Novikova et al., 2010). MccE belongs to the general control non-repressible 5-related *N*-acetyltransferases (GNAT) superfamily, and shows high similarity with chromosomally encoded acetyltransferases RimI, RimJ, and RimL, which acetylate the N-termini of ribosomal proteins S18, S5, and L12 (Salah Ud-Din et al., 2016). Indeed, *E. coli* RimL induces resistance to McC by acetylating the amino group of the processed McC aspartate by the same mechanism as MccE (Kazakov et al., 2014). Similarly, when overproduced, RimL makes cells resistant to albomycin by acetylating processed albomycin, which contains a pyrimidine nucleotide instead of adenosine. Subsequently, a potential cross-resistance between McC and albomycin is quite possible (Kazakov et al., 2014). The MccF serine protease hydrolyses the carboxamide bond between the C-terminal aspartamide and AMP of both intact and processed McC, thus inactivating the aspartyl-adenylate (Agarwal et al., 2012). Moreover, McC inactivation is also ensured by phosphoramidases belonging to the histidine-triad (HIT) superfamily hydrolases that can either be encoded in certain *mcc*-like biosynthetic clusters or by genes located elsewhere in bacterial genomes (Yagmurov et al., 2020). Resistance to McC-like compounds produced by *S. enterica*, *Nocardiopsis kunsanensis*, *P. fluorescens* or *Hyalangium minutum* is conferred by hydrolysis of the phosphoramide bond in the toxic aspartamide-adenylate (Yagmurov et al., 2020).Therefore, it appears that resistance to McC and McC-like microcins by toxin inactivation can occur via both enzymes encoded in the microcin biosynthesis clusters and more generalist and non-specific enzymes sharing structural similarities.

Finally, impairment of the final three-dimensional structure of the antibacterial peptide, such as by preventing the formation of disulfide bridges, could be a last mechanism resulting in resistance to microcins. This has been poorly explored until now, but is however illustrated by MccPDI for which mutations in *dsbA*, *dsbB* genes induce resistance to MccPDI (Zhao et al., 2015). Genes *dsbA*, *dsbB* encode DsbA and DsbB thiol-redox enzymes that usually catalyze disulfide bond formation for proteins that are transported into the periplasm, and which would be possibly involved in formation of the disulfide bond that stabilizes this microcin.

## Inhibitory Effect of Microcins Against Antibiotic Resistant Gram-Negative Bacteria

The spectrum of inhibitory activity of microcins includes a wide number of bacteria which are phylogenetically related to the producing strain including *Salmonella, Shigella* and *E. coli*. The inhibition activity of the different microcins against non-multidrug-resistant strains has been reported in the literature. However, the potency of these microcins specifically against MDR bacteria has not been systematically described and only few studies have addressed this special issue.

MccJ25 was shown to exhibit a high antimicrobial activity against MDR *Salmonella* and *E. coli* (Martin-Gómez et al., 2019; Yu et al., 2019). The antimicrobial activity of MccJ25 was also extensively studied against a collection of MDR strains of *S. enterica* spp. *enterica* (Ben Said et al., 2020). Interestingly, this study has shown that *Salmonella* strains exhibit various sensitivity profiles to MccJ25 and that MIC values vary from 0.06 to 400 μg/mL (0.028–189 nM), independently of the resistance profiles to antibiotics or the serovars. Other studies have shown that MccJ25 displays a great inhibitory potential against *Salmonella* and *E. coli* (Sablé et al., 2000; Delgado et al., 2001; Rintoul et al., 2001; Soudy et al., 2012). MccPDI is known to inhibit foodborne pathogenic enterohemorrhagic *E. coli* serotypes O157:H7 and O26 (Eberhart et al., 2012) as well as *Shigella* strains and *E. coli* isolates that are MDR strains (Lu et al., 2019). Likewise, MccH47 has demonstrated a potent effect against *Enterobacteriaceae* MDR strains including *Salmonella* and *E. coli* carbapenemase, extended spectrum β-lactamase and metallo-β-lactamase producers. MccH47 has MIC values less than 75 μg/mL (13 μM) for all tested strains (Palmer et al., 2020).

The remaining microcins revealed similar narrow spectra of activity against non-MDR *Enterobacteriaceae*, mainly *Salmonella* and *E. coli.* Indeed, MccE492 was shown to have inhibitory activity *in vitro* against a wide range of *Enterobacteriaceae* including *Klebsiella*, *Enterobacter*, *E. coli* and *Salmonella* while MccM was shown to inhibit *Salmonella* and *E. coli* (Vassiliadis et al., 2010). MccN/24 is active against *E. coli* and *S. enterica* Typhimurium, but not against *L. monocytogenes* or *Campylobacter jejuni* (Wooley et al., 1999). It was also reported by Kaur et al. (2016) that MccN/24 exhibits a potent activity against *Salmonella* strains. Furthermore, MccV is active against some pathogenic *E. coli* with MIC values ranging from 7.7 × 10^–3^ to 13.25 μg/mL (0.89–1517.94 nM) (Boubezari et al., 2018). MccS is lethal to virulent enterohemorrhagic and enteropathogenic *E. coli* through inhibiting the adherence of EPEC *E. coli* to intestinal epithelial cells in an *in vitro* adherence assay (Zschüttig et al., 2012). MccL exhibits a strong antibacterial activity against *Enterobacteriaceae*, including the *S. enterica* serovars Typhimurium and Enteritidis (Morin et al., 2011).

Only a few studies have systematically assessed the efficiency of microcins (and, more generally, of bacteriocins), for the inhibition of MDR bacteria, and/or the microcin/bacteriocin and antibiotic cross-resistance (Ben Said et al., 2020; Kuznetsova et al., 2020). Although the activities reported so far are encouraging, more systematic studies on the inhibitory potential of microcins against MDR strains remain necessary to confirm the potential of microcins as alternatives to antibiotics against MDR and are thus of high research priority. Future directions of research should relate to both qualitative and quantitative *in vitro* characterization of the inhibitory activity of different microcins against a large panel of clinical isolates of MDR pathogenic bacteria of medical and veterinary interest, coming from well characterized reference collections. The development of resistance of these strains against the various microcins deserves being invertigated as well as studying the possible synergistic effects between microcins and certain antibiotics or biocides, as already started with Gram-positive bacteriocins (Mathur et al., 2017). Indeed, the identification of compounds with synergistic or additive effects could represent an effective strategy to limit the development of bacteria resistant to both microcins and antibiotics. Such an approach, and more widely combination treatment therapeutic strategies, could be facilitated by the development of optimized methods to quantify synergy effects more rapidly and efficiently (Fatsis-Kavalopoulos et al., 2020).

## Microcins and the Immune System

Inflammation is one of the key processes allowing the immune system being alerted of risks for the host, such as pathogen attacks. But its dysregulation results in chronic inflammation and subsequent diseases, pointing that inflammation results in both beneficial and adverse effects. In general, interactions of bacteriocins or microcins with the immune system have not been investigated deeply, which hampers evaluating previsible risks and benefits for all characterized microcins. MccE492 was reported to induce apoptosis against human cell lines without inducing an inflammatory response (Hetz et al., 2002; Lagos et al., 2009). But most of all, two microcins, MccB17 and especially MccJ25, have been chiefly studied in this regard.

A pionneer study showed that polyclonal antibodies were raised in rabbits against mature MccB17, indicating that it could induce immune reaction once introduced in host body (Yorgey et al., 1993). In an in-depth study on the effects of oxazole compounds on intestinal inflammation (Iyer et al., 2018) have shown that, similar to environmental or synthetic ones, short-size oxazole compounds derived from MccB17 degradation were able to induce inflammation in mouse intestinal epithelial cells, while full-length MccB17 was not (Iyer et al., 2018; Collin and Maxwell, 2019). This effect was attributed to a cascade response where oxazole compounds activate IDO1, the rate-limiting enzyme in tryptophan catabolism, and in turn tryptophan-derived metabolites activate the aryl hydrocarbon receptor Ahr, which limits CD1d-restricted production of the anti-inflammatory cytokine IL-10 and results in natural killer T-cell mediated intestinal inflammation (Iyer et al., 2018). It was pointed that this oxazole-induced intestinal inflammation is independent of the antimicrobial activity of the compounds. Moreover, it was proposed that the CD1d-dependent immunomodulatory effect is limited by the size of the compounds, explaining the absence of effect of native MccB17, although its content in oxazole rings.

An *in vitro* study showed that MccJ25 protects IPEC-J2 cells against enterotoxigenic *E. coli* (ETEC) without raising cytotoxicity and alleviates the inflammatory responses through modulation of the levels of pro-inflammatory cytokines, interleukins 6 (IL-6), IL-8 and tumor necrosis factor-α (TNF-α) (Yu et al., 2018a). An anti-inflammatory effect of MccJ25 associated with killing of the pathogen was shown in an ETEC-infected mouse model (Ding et al., 2020; Yu et al., 2020). Similar to gentamicin treated mice, the levels of pro-inflammatory cytokines were significantly decreased in jejunum, ileum and colon tissues of mice administered MccJ25, compared to the control group, while the anti-inflammatory IL-10 level increased. Inhibition of ETEC-induced expression of inflammatory cytokines in the jejunum was proposed to be due to down-regulation by MccJ25 of the NF-κB and mitogen-activated protein kinase (MAPK) pathways (Ding et al., 2020). Moreover, absence of immunomodulatory effect and toxicity of MccJ25 was observed at the therapeutic dose (9 mg/kg), much higher doses only (18 mg/kg) being able to cause a low toxicity (Yu et al., 2018b). Furthermore, MccJ25 also decreases the serum concentration levels of the pro-inflammatory cytokines IL-6, IL-1β, and TNF-α, together with an increase in anti-inflammatory IL-10 in weaned pigs (Wang et al., 2020) and in broiler chicken (Yu et al., 2017) fed with MccJ25-supplemented diet. Taken together, these *in vivo* studies conducted in different animal models indicate that MccJ25 diet supplementation can lower inflammation together with affording protection against pathogens, providing interesting perspectives in inflammatory intestinal diseases. Therefore, it appears that none of the studied microcins appears to induce adverse inflammation imbalance and have a detrimental effect on the host.

## Potential Applications of Microcins and Future Prospect

Microcins exhibit a number of advantages for potential applications, among which their absence of toxicity to eukaryotic cells and their chemical stability. Indeed, the three-dimensional structures or PTMs of most microcins increases their stability to harsh conditions, such as those that are encountered in the gut (Naimi et al., 2020). This favors their delivery to the gut without the help of specific formulations, if not for avoiding immunity response. However, unfortunately, the spectrum of inhibitory activity of the different microcins has not been deeply investigated, hampering significant development in veterinary or human medical domains. The antimicrobial activity of most microcins (MccB17, McC and a few others) was determined in order to decipher their mechanism of action and the most tested bacterium was *E. coli* (Heddle et al., 2001; Metlitskaya et al., 2006; Severinov and Nair, 2012). Thus, while for a few microcins the spectrum of inhibition is well known, for the remaining this information is still missing. A more systematic study involving a significant number of clinical and veterinary pathogens, including MDR strains, remains necessary to establish the exact spectrum of inhibition of each microcin.

An important characteristic making microcins good candidates as alternatives to antibiotics is that they are prominent actors of competitions in microbiota and particularly in the gut microbiota, which is the most studied. Microcins play a significant role in niche competition (for a review see Li and Rebuffat, 2020), essentially in interference competition, which involves the secretion of harmful molecules such as the microcins, for direct attack of competitors. But also in a lesser extent, they are involved in the indirect process of exploitative competition, as exemplified by siderophore microcins which are able to capture iron and thus deplete the surroundings of this essential element. Thereby, the siderophore microcins MccH47 and MccM, both produced by the probiotic *E. coli* strain Nissle 1917, have been shown to mediate competition among *Enterobacteriaceae* in mouse model and to impair the growth of the pathogen *S. enterica* serovar Typhimurium in the inflamed gut, where iron is scarce, without perturbing significantly the microbiota equilibrium (Sassone-Corsi et al., 2016). Thanks to their natural role in their niche, which involves both high potency and narrow spectrum of activity, the molecules from microbiota, such as the microcins in the gut microbiota (Donia and Fischbach, 2015; Garcia-Gutierrez et al., 2019), or other bacteriocins in the rumen (Oyama et al., 2017), are thus of high potential. However, exploration of the capacity of microorganisms belonging to various microbiota still remains underdeveloped so far. Its development in combination with genome mining approaches and innovative computational technologies should allow finding novel microcins, and possibly novel mechanisms of action.

To explore the potential applications of microcins in animal and human health, *in vivo* studies have been conducted, although they are still few and only concern a few microcins, essentially MccJ25. For instance, a significant decrease of *S.* Typhimurium was recorded in chicken, using an *E. coli* transformant strain producing MccN/24, although continuous administration of the transformant was needed to ensure colonization within the *in vivo* model (Wooley et al., 1999). MccJ25 has been shown to decrease *S. enterica* counts in the liver and spleen in mice (Lopez et al., 2007) and in the gastrointestinal tract of turkeys (Forkus et al., 2017), and to relieve diarrhea and systematic inflammation in weaned pigs (Yu et al., 2017). Furthermore, MccJ25 was shown to improve performance, fecal microbiota composition and systematic inflammation of broilers (Wang et al., 2020). Further studies are needed however to validate the potential of microcins as therapeutic agents in human or veterinary medicine.

Finally, developing safe probiotics engineered to produce potent microcins is a complementary and efficient approach. It relies on previous studies of commercially available probiotics, *E. coli* Nissle 1917 (Mutaflor^®^) and *E. coli* G3/10 (Symbioflor2^®^), producers of microcins MccH47 and MccM (Sassone-Corsi et al., 2016; Massip and Oswald, 2020) and MccS (Zschüttig et al., 2012), respectively, which were shown to act in bacterial competition and kill pathogens in inflamed gut (Sassone-Corsi et al., 2016), or suppress adherence of enteropathogenic *E. coli* (Zschüttig et al., 2012). Thus, *S. enterica* carriage was significantly reduced in turkey gastrointestinal tract using *E. coli* Nissle engineered to produce MccJ25 (Forkus et al., 2017). Furthermore, *E. coli* Nissle was engineered to produce MccH47 in response to tetrathionate, which is produced in gut inflammation conditions and is favorable to *Salmonella* growth (Palmer et al., 2018). In this system, MccH47 was produced in response to the tetrathionate environmental signal serving as an inducing molecule, and inhibited the pathogen *S.* Typhimurium, both in static inhibition assays and in ecological competition experiments.

## Conclusion

As it can be seen through this review, microcins offer an attractive track for designing novel antimicrobial strategies and envisage alternatives to conventional antibiotics, despite the potential risks of resistance, cross-resistance and co-resistance that have been pointed. The microcin attractivity relies first on their two-step mechanisms of action. The first step ensures uptake of the microcin and involves most often a Trojan horse strategy. It is exemplarily illustrated by MccC, for which the last processing step of the uptaken harmless nucleotide peptide is ensured in the targeted bacteria by common proteases. It is also exemplified by siderophore microcins (MccE492, MccM, MccH47) or the lasso microcin MccJ25 that mimic the natural ligands of siderophore receptors to hijack them. The second step implies either membrane perturbations or inhibition of critical enzymes, and therefore vital functions in bacteria. Indeed, in certain cases such strategies are shared by antibiotics, which can result in cross-resistance, as pointed in this review. These two steps can also constitute a drawback toward resistance development as inhibiting one of them could potentially confer resistance to microcin. However, a few microcins, such as McC and MccJ25, bring into play a second and independent mechanism that intervenes at higher concentrations. Such a secondary mechanism has not been brought to light for other microcins, but it must be said that it has not been thoroughly investigated. Such a succession of different mechanisms limits the emergence of bacterial resistance, as the energetic costs induced by setting up distinct resistance mechanisms simultaneously is hard to assume by the bacteria.

Other characteristics, which have been underlined in the review, support their interesting potential: (i) a potent activity in the GI tract, (ii) a narrow spectrum of activity, which makes them active against pathogens while preserving host microbiota, (iii) an important role in microbial competitions, which makes them actors in maintaining microbiota equilibrium, (iv) an efficient activity *in vivo* in different animal models. Developing strategies based on Nature-derived mechanisms and molecules that are able to minimize both niche perturbations and resistance thus appears as a promising direction in the light of recent analysis of the frequency and mechanisms of resistance of antimicrobial peptides and antibiotics (Kintses et al., 2019). Finally, as the production costs of antimicrobial peptides and in particular of RiPPs remain high, a possible strategy to use microcins and simultaneously increase their potency could be to associate them to conventional antibiotics. This would take full advantage of the lower costs of production of antibiotics, of an increased potency when synergistic effects are obtained, and of the possibility of combining distinct mechanisms of action. Therefore, relying on the current knowledge on the topology of microcins and their targets, the microcin biosynthesis pathways, and their mechanisms of action and of resistance, directions of research involving a more dynamic exploration of diverse microbiota associated with the development of microcin bioengineering would presumably accelerate the diversification of anti-AMR strategies.

## Author Contributions

All authors listed have made a substantial, direct and intellectual contribution to the work, approved the final manuscript for publication. ST and LBS drafted the manuscript and contributed equally to its preparation. SR, IF, and SZ revised the manuscript. LBS and SR wrote the final version of the manuscript. ST, LBS, and SZ designed and prepared the figures.

## Conflict of Interest

The authors declare that the research was conducted in the absence of any commercial or financial relationships that could be construed as a potential conflict of interest.

## References

[B1] Adelman K. Yuzenkova J. La Porta A. Zenkin N. Lee J. Lis J. T. (2004). Molecular mechanism of transcription inhibition by peptide antibiotic Microcin J25. *Mol. Cell.* 14 753–762. 10.1016/j.molcel.2004.05.017 15200953

[B2] Agarwal V. Metlitskaya A. Severinov K. Nair S. K. (2011). Structural basis for microcin C7 inactivation by the MccE acetyltransferase. *J. Biol. Chem.* 286 21295–21303. 10.1074/jbc.M111.226282 21507941 PMC3122189

[B3] Agarwal V. Tikhonov A. Metlitskaya A. Severinov K. Nair S. K. (2012). Structure and function of a serine carboxypeptidase adapted for degradation of the protein synthesis antibiotic microcin C7. *Proc. Natl. Acad. Sci. U.S.A.* 109 4425–4430. 10.1073/pnas.1114224109 22388748 PMC3311384

[B4] Aguilera P. Marcoleta A. Lobos-Ruiz P. Arranz R. Valpuesta J. M. Monasterio O. (2016). Identification of key amino acid residues modulating intracellular and in vitro microcin E492 amyloid formation. *Front. Microbiol.* 7:35. 10.3389/fmicb.2016.00035 26858708 PMC4729943

[B5] Amaral L. Martins A. Spengler G. Molnar J. (2014). Efflux pumps of Gram-negative bacteria, what they do, how they do it, with what and how to deal with them. *Front. Pharmacol.* 4:168. 10.3389/fphar.2013.00168 24427138 PMC3879458

[B6] Anes J. McCusker M. P. Fanning S. Martins M. (2015). The ins and outs of RND efflux pumps in *Escherichia coli*. *Front. Microbiol.* 6:587. 10.3389/fmicb.2015.00587 26113845 PMC4462101

[B7] Arnison P. G. Bibb M. J. Bierbaum G. Bowers A. A. Bugni T. S. Bulaj G. (2013). Ribosomally synthesized and post-translationally modified peptide natural products, overview and recommendations for a universal nomenclature. *Nat. Prod. Rep.* 30 108–160. 10.1039/c2np20085f 23165928 PMC3954855

[B8] Arranz R. Mercado G. Martin-Benito J. Giraldo R. Monasterio O. Lagos R. (2012). Structural characterization of microcin E492 amyloid formation, Identification of the precursors. *J. Struct. Biol.* 178 54–60. 10.1016/j.jsb.2012.02.015 22420976

[B9] Asensio C. Perez-Diaz J. C. (1976). A new family of low molecular weight antibiotics from enterobacteria. *Biochem. Biophys. Res. Commun.* 69 7–14. 10.1016/s0006-291x(76)80264-1 4071

[B10] Azpiroz M. F. Laviña M. (2007). Modular structure of microcin H47 and colicin V. *Antimicrob. Agents Chemother.* 51 2412–2419. 10.1128/AAC.01606-06 17452478 PMC1913283

[B11] Bantysh O. Serebryakova M. Makarova K. S. Dubiley S. Datsenko K. A. Severinov K. (2014). Enzymatic synthesis of bioinformatically predicted microcin C-like compounds encoded by diverse bacteria. *mBio* 5:e01059-14. 10.1128/mBio.01059-14 24803518 PMC4010828

[B12] Baquero F. Lanza V. F. Baquero M. R. Del Campo R. Bravo-Vazquez D. A. (2019). Microcins in *Enterobacteriaceae*, Peptide antimicrobials in the eco-active intestinal chemosphere. *Front. Microbiol.* 10:2261. 10.3389/fmicb.2019.02261 31649628 PMC6795089

[B13] Baquero F. Moreno F. (1984). The microcins. *FEMS Microbiol. Lett.* 23 117–124. 10.1007/978-3-642-76974-0_12

[B14] Beis K. Rebuffat S. (2019). Multifaceted ABC transporters associated to microcin and bacteriocin export. *Res. Microbiol.* 170 399–406. 10.1016/j.resmic.2019.07.002 31401108

[B15] Bellomio A. Vincent P. A. de Arcuri B. F. Farias R. N. Morero R. D. (2007). Microcin J25 has dual and independent mechanisms of action in *Escherichia coli*, RNA polymerase inhibition and increased superoxide production. *J. Bacteriol.* 189 4180–4186. 10.1128/jb.00206-07 17400747 PMC1913388

[B16] Ben Said L. Emond-Rheault J. G. Soltani S. Telhig S. Zirah S. Rebuffat S. (2020). Phenomic and genomic approaches to studying the inhibition of multiresistant *Salmonella enterica* by microcin J25. *Environ. Microbiol*. 22 2907–2920. 10.1111/1462-2920.15045 32363677

[B17] Bieler S. Estrada L. Lagos R. Baeza M. Castilla J. Soto C. (2005). Amyloid formation modulates the biological activity of a bacterial protein. *J. Biol. Chem.* 280 26880–26885. 10.1074/jbc.M502031200 15917245

[B18] Bieler S. Silva F. Belin D. (2010). The polypeptide core of microcin E492 stably associates with the mannose permease and interferes with mannose metabolism. *Res. Microbiol.* 161 706–710. 10.1016/j.resmic.2010.07.003 20674740

[B19] Bieler S. Silva F. Soto C. Belin D. (2006). Bactericidal activity of both secreted and nonsecreted microcin E492 requires the mannose permease. *J. Bacteriol.* 188 7049–7061. 10.1128/jb.00688-06 17015644 PMC1636244

[B20] Boubezari M. T. Idoui T. Hammami R. Fernandez B. Gomaa A. Fliss I. (2018). Bacteriocinogenic properties of *Escherichia coli* P2C isolated from pig gastrointestinal tract, purification and characterization of microcin V. *Arch. Microbiol.* 200 771–782. 10.1007/s00203-018-1482-6 29417164

[B21] Bountra K. Hagelueken G. Choudhury H. G. Corradi V. El Omari K. Wagner A. (2017). Structural basis for antibacterial peptide self-immunity by the bacterial ABC transporter McjD. *Embo. J.* 36 3062–3079. 10.15252/embj.201797278 28864543 PMC5641919

[B22] Braffman N. R. Piscotta F. J. Hauver J. Campbell E. A. Link A. J. Darst S. A. (2019). Structural mechanism of transcription inhibition by lasso peptides microcin J25 and capistruin. *Proc. Natl. Acad. Sci. U.S.A.* 116 1273–1278. 10.1073/pnas.1817352116 30626643 PMC6347699

[B23] Brown E. E. F. Cooper A. Carrillo C. Blais B. (2019). Selection of multidrug-resistant bacteria in medicated animal feeds. *Front. Microbiol.* 10:456. 10.3389/fmicb.2019.00456 30894847 PMC6414793

[B24] Budic M. Rijavec M. Petkovsek Z. Zgur-Bertok D. (2011). *Escherichia coli* bacteriocins, antimicrobial efficacy and prevalence among isolates from patients with bacteraemia. *PLoS One* 6:e28769. 10.1371/journal.pone.0028769 22205967 PMC3242755

[B25] Burkhart B. J. Hudson G. A. Dunbar K. L. Mitchell D. A. (2015). A prevalent peptide-binding domain guides ribosomal natural product biosynthesis. *Nat. Chem. Biol.* 11 564–570. 10.1038/nchembio.1856 26167873 PMC4509860

[B26] Bush K. Jacoby G. A. (2010). Updated functional classification of beta-lactamases. *Antimicrob. Agents Chemother.* 54 969–976. 10.1128/aac.01009-09 19995920 PMC2825993

[B27] Calvopina K. Dulyayangkul P. Heesom K. J. Avison M. B. (2020). TonB-dependent uptake of beta-lactam antibiotics in the opportunistic human pathogen *Stenotrophomonas maltophilia*. *Mol. Microbiol.* 113 492–503. 10.1111/mmi.14434 31773806

[B28] Campbell E. A. Korzheva N. Mustaev A. Murakami K. Nair S. Goldfarb A. (2001). Structural mechanism for rifampicin inhibition of bacterial RNA polymerase. *Cell* 104 901–912. 10.1016/s0092-8674(01)00286-0 11290327

[B29] Campos M. A. Vargas M. A. Regueiro V. Llompart C. M. Albertí S. Bengoechea J. A. (2004). Capsule polysaccharide mediates bacterial resistance to antimicrobial peptides. *Infect. Immun.* 72 7107–7114. 10.1128/IAI.72.12.7107-7114.2004 15557634 PMC529140

[B30] Carlson S. A. Frana T. S. Griffith R. W. (2001). Antibiotic resistance in *Salmonella enterica* serovar Typhimurium exposed to microcin-producing *Escherichia coli*. *Appl. Environ. Microbiol.* 67 3763–3766. 10.1128/aem.67.8.3763-3766.2001 11472964 PMC93088

[B31] Carroll L. M. Gaballa A. Guldimann C. Sullivan G. Henderson L. O. Wiedmann M. (2019). Identification of novel mobilized colistin resistance gene mcr-9 in a multidrug-resistant, colistin-susceptible *Salmonella enterica* serotype Typhimurium isolate. *mBio* 10:e0853-19. 10.1128/mBio.00853-19 31064835 PMC6509194

[B32] Cascales E. Buchanan S. K. Duché D. Kleanthous C. Lloubès R. Postle K. (2007). Colicin biology. *Microbiol. Mol. Biol. Rev.* 71 158–229. 10.1128/mmbr.00036-06 17347522 PMC1847374

[B33] Chapman J. S. (2003). Disinfectant resistance mechanisms, cross-resistance, and co-resistance. *Int. Biodeterior. Biodegrad.* 51 271–276. 10.1016/S0964-8305(03)00044-1

[B34] Chehade H. Braun V. (1988). Iron-regulated synthesis and uptake of colicin V. *FEMS Microbiol. Lett.* 52 177–181. 10.111/j.1574-6968.1988.tb02591.x

[B35] Cheung-Lee W. L. Link A. J. (2019). Genome mining for lasso peptides, past, present, and future. *J. Ind. Microbiol. Biot*. 46 1371–1379. 10.1007/s10295-019-02197-z 31165971 PMC6989040

[B36] Cheung-Lee W. L. Parry M. E. Cartagena A. J. Darst S. A. Link A. J. (2019). Discovery and structure of the antimicrobial lasso peptide citrocin. *J. Biol. Chem*. 294 6822–6830. 10.1074/jbc.RA118.006494 30846564 PMC6497930

[B37] Cheung-Lee W. L. Parry M. E. Zong C. Cartagena A. J. Darst S. A. Connell N. D. (2020). Discovery of ubonodin, an antimicrobial lasso peptide active against members of the *Burkholderia cepacia* complex. *Chembiochem*. 21 1335–1340. 10.1002/cbic.201900707 31765515 PMC7205569

[B38] Choi U. Lee C. R. (2019). Distinct roles of outer membrane porins in antibiotic resistance and membrane integrity in *Escherichia coli*. *Front. Microbiol*. 10:953. 10.3389/fmicb.2019.00953 31114568 PMC6503746

[B39] Chopra I. Roberts M. (2001). Tetracycline antibiotics, mode of action, applications, molecular biology, and epidemiology of bacterial resistance. *Microbiol. Mol. Biol. Rev.* 65 232–260. 10.1128/mmbr.65.2.232-260.2001 11381101 PMC99026

[B40] Collet J.-F. Cho S.-H. Iorga B. I. Goemans C. V. (2020). How the assembly and protection of the bacterial cell envelope depend on cysteine residues. *J. Biol. Chem.* 295 11984–11994. 10.1074/jbc.REV120.011201 32487747 PMC7443483

[B41] Collin F. Maxwell A. (2019). The microbial toxin Microcin B17, prospects for the development of new antibacterial agents. *J. Mol. Biol.* 431 3400–3426. 10.1016/j.jmb.2019.05.050 31181289 PMC6722960

[B42] Corsini G. Karahanian E. Tello M. Fernandez K. Rivero D. Saavedra J. M. (2010). Purification and characterization of the antimicrobial peptide microcin N. *FEMS Microbiol. Lett.* 312 119–125. 10.1111/j.1574-6968.2010.02106.x 20979348

[B43] Cotter P. D. Ross R. P. Hill C. (2013). Bacteriocins - a viable alternative to antibiotics? *Nat. Rev. Microbiol*. 11 95–105. 10.1038/nrmicro2937 23268227

[B44] Crosa J. H. Walsh C. T. (2002). Genetics and assembly line enzymology of siderophore biosynthesis in bacteria. *Microbiol. Mol. Biol. Rev.* 66 223–249. 10.1128/mmbr.66.2.223-249.2002 12040125 PMC120789

[B45] Davies E. A. Bevis H. E. Delves-Broughton J. (1997). The use of the bacteriocin, nisin, as a preservative in ricotta-type cheeses to control the food-borne pathogen *Listeria monocytogenes*. *Lett. Appl. Microbiol*. 24 343–346. 10.1046/j.1472-765x.1997.00145.x 9172439

[B46] de Kraker M. E. Stewardson A. J. Harbarth S. (2016). Will 10 million people die a year due to antimicrobial resistance by 2050? *PLoS Med*. 13:e1002184. 10.1371/journal.pmed.1002184 27898664 PMC5127510

[B47] de Lorenzo V. (1984). Isolation and characterization of microcin E 492 from *Klebsiella pneumoniae*. *Arch. Microbiol.* 139 72–75. 10.1007/BF00692715 6385903

[B48] Deegan L. H. Cotter P. D. Hill C. Ross P. (2006). Bacteriocins, biological tools for bio-preservation and shelf-life extension. *Int. Dairy J.* 16 1058–1071. 10.1016/j.idairyj.2005.10.026

[B49] del Castillo F. J. del Castillo I. Moreno F. (2001). Construction and characterization of mutations at codon 751 of the *Escherichia coli* gyrB gene that confer resistance to the antimicrobial peptide microcin B17 and alter the activity of DNA gyrase. *J. Bacteriol.* 183 2137–2140. 10.1128/jb.183.6.2137-2140.2001 11222617 PMC95114

[B50] Delgado M. A. Rintoul M. R. Farias R. N. Salomón R. A. (2001). *Escherichia coli* RNA polymerase is the target of the cyclopeptide antibiotic microcin J25. *J. Bacteriol.* 183 4543–4550. 10.1128/jb.183.15.4543-4550.2001 11443089 PMC95349

[B51] Delgado M. A. Vincent P. A. Farias R. N. Salomón R. A. (2005). YojI of *Escherichia coli* functions as a microcin J25 efflux pump. *J. Bacteriol.* 187 3465–3470. 10.1128/jb.187.10.3465-3470.2005 15866933 PMC1112001

[B52] Destoumieux-Garzón D. Duquesne S. Peduzzi J. Goulard C. Desmadril M. Letellier L. (2005). The iron-siderophore transporter FhuA is the receptor for the antimicrobial peptide microcin J25, role of the microcin Val11-Pro16 beta-hairpin region in the recognition mechanism. *Biochem. J.* 389 869–876. 10.1042/bj20042107 15862112 PMC1180738

[B53] Destoumieux-Garzón D. Peduzzi J. Thomas X. Djediat C. Rebuffat S. (2006). Parasitism of iron-siderophore receptors of *Escherichia coli* by the siderophore-peptide microcin E492m and its unmodified counterpart. *Biometals* 19 181–191. 10.1007/s10534-005-4452-9 16718603

[B54] Destoumieux-Garzón D. Thomas X. Santamaria M. Goulard C. Barthélémy M. Boscher B. (2003). Microcin E492 antibacterial activity, evidence for a TonB-dependent inner membrane permeabilization on *Escherichia coli*. *Mol. Microbiol.* 49 1031–1041. 10.1046/j.1365-2958.2003.03610.x 12890026

[B55] Dickey S. W. Cheung G. Y. C. Otto M. (2017). Different drugs for bad bugs, antivirulence strategies in the age of antibiotic resistance. *Nat. Rev. Drug Discov.* 16 457–471. 10.1038/nrd.2017.23 28337021 PMC11849574

[B56] Dijkmans A. C. Zacarias N. V. O. Burggraaf J. Mouton J. W. Wilms E. B. van Nieuwkoop C. (2017). Fosfomycin, pharmacological, clinical and future perspectives. *Antibiotics* 6:24. 10.3390/antibiotics6040024 29088073 PMC5745467

[B57] Ding X. Yu H. Qiao S. (2020). Lasso peptide microcin J25 effectively enhances gut barrier function and modulates inflammatory response in an enterotoxigenic *Escherichia coli*-challenged mouse model. *Int. J. Mol. Sci.* 21:E6500. 10.3390/ijms21186500 32899529 PMC7555725

[B58] Doi Y. Bonomo R. A. Hooper D. C. Kaye K. S. Johnson J. R. Clancy C. J. (2017). Gram-negative bacterial infections, research priorities, accomplishments, and future directions of the antibacterial resistance leadership group. *Clin. Infect. Dis.* 64 S30–S35. 10.1093/cid/ciw829 28350901 PMC5848311

[B59] Donia M. S. Fischbach M. A. (2015). Human Microbiota. Small molecules from the human microbiota. *Science* 349:1254766. 10.1126/science.1254766 26206939 PMC4641445

[B60] Drider D. Rebuffat S. (2011). *Antimicrobial Peptides, From Genes to Applications.* New York, NY: Springer-Verlag New York.

[B61] Drissi F. Buffet S. Raoult D. Merhej V. (2015). Common occurrence of antibacterial agents in human intestinal microbiota. *Front. Microbiol.* 6:441. 10.3389/fmicb.2015.00441 25999943 PMC4423438

[B62] Duquesne S. Destoumieux-Garzón D. Peduzzi J. Rebuffat S. (2007a). Microcins, gene-encoded antibacterial peptides from enterobacteria. *Nat. Prod. Rep.* 24 708–734. 10.1039/b516237h 17653356

[B63] Duquesne S. Destoumieux-Garzón D. Zirah S. Goulard C. Peduzzi J. Rebuffat S. (2007b). Two enzymes catalyze the maturation of a lasso peptide in *Escherichia coli*. *Chem. Biol.* 14 793–803. 10.1016/j.chembiol.2007.06.004 17656316

[B64] Eberhart L. J. Deringer J. R. Brayton K. A. Sawant A. A. Besser T. E. Call D. R. (2012). Characterization of a novel microcin that kills enterohemorrhagic *Escherichia coli* O157, H7 and O26. *Appl. Environ. Microbiol.* 78 6592–6599. 10.1128/AEM.01067-12 22773653 PMC3426703

[B65] Egan K. Ross R. P. Hill C. (2017). Bacteriocins, antibiotics in the age of the microbiome. *Emerging Top Life Sci.* 1 55–63. 10.1042/ETLS20160015 33525813

[B66] Endriss F. Braun M. Killmann H. Braun V. (2003). Mutant analysis of the *Escherichia coli* FhuA protein reveals sites of FhuA activity. *J. Bacteriol.* 185 4683–4692. 10.1128/jb.185.16.4683-4692.2003 12896986 PMC166461

[B67] Fabrega A. Madurga S. Giralt E. Vila J. (2009). Mechanism of action of and resistance to quinolones. *Microb. Biotechnol.* 2 40–61. 10.1111/j.1751-7915.2008.00063.x 21261881 PMC3815421

[B68] Falagas M. E. Athanasaki F. Voulgaris G. L. Triarides N. A. Vardakas K. Z. (2019). resistance to fosfomycin, mechanisms, frequency and clinical consequences. *Int. J. Antimicrob. Agents* 53 22–28. 10.1016/j.ijantimicag.2018.09.013 30268576

[B69] Fath M. J. Zhang L. H. Rush J. Kolter R. (1994). Purification and characterization of colicin V from *Escherichia coli* culture supernatants. *Biochemistry* 33, 6911–6917. 10.1021/bi00188a021 8204625

[B70] Fatsis-Kavalopoulos N. Roemhild R. Tang P.-C. Kreuger J. Andersson D. I. (2020). CombiANT, antibiotic interaction testing made easy. *PLoS Biol.* 18:e3000856. 10.1371/journal.pbio.3000856 32941420 PMC7524002

[B71] Forkus B. Ritter S. Vlysidis M. Geldart K. Kaznessis Y. N. (2017). Antimicrobial probiotics reduce *Salmonella enterica* in turkey gastrointestinal tracts. *Sci. Rep.* 7:40695. 10.1038/srep40695 28094807 PMC5240571

[B72] Garcia-Gutierrez E. Mayer M. J. Cotter P. D. Narbad A. (2019). Gut microbiota as a source of novel antimicrobials. *Gut Microb.* 10 1–21. 10.1080/19490976.2018.1455790 29584555 PMC6363078

[B73] Gerard F. Pradel N. Wu L. F. (2005). Bactericidal activity of colicin V is mediated by an inner membrane protein, SdaC, of *Escherichia coli*. *J. Bacteriol.* 187 1945–1950. 10.1128/jb.187.6.1945-1950.2005 15743941 PMC1064040

[B74] Ghai I. Ghai S. (2018). Understanding antibiotic resistance via outer membrane permeability. *Infect. Drug Resist.* 11 523–530. 10.2147/IDR.S156995 29695921 PMC5903844

[B75] Ghilarov D. Serebryakova M. Stevenson C. E. M. Hearnshaw S. J. Volkov D. S. Maxwell A. (2017). The origins of specificity in the microcin-processing protease TldD/E. *Structure* 25 1549–1561.e5. 10.1016/j.str.2017.08.006 28943336 PMC5810440

[B76] Ghilarov D. Stevenson C. E. M. Travin D. Y. Piskunova J. Serebryakova M. Maxwell A. (2019). Architecture of microcin B17 synthetase. An octameric protein complex converting a ribosomally synthesized peptide into a DNA gyrase poison. *Mol. Cell.* 73 749–762.e5. 10.1016/j.molcel.2018.11.032 30661981 PMC6395948

[B77] Ghosh C. Sarkar P. Issa R. Haldar J. (2019). Alternatives to conventional antibiotics in the era of antimicrobial resistance. *Trends Microbiol.* 27 323–338. 10.1016/j.tim.2018.12.010 30683453

[B78] Gibson M. K. Wang B. Ahmadi S. Burnham C. A. Tarr P. I. Warner B. B. (2016). Developmental dynamics of the preterm infant gut microbiota and antibiotic resistome. *Nat. Microbiol.* 1:16024. 10.1038/nmicrobiol.2016.24 27572443 PMC5031140

[B79] Goldstein B. P. (2014). Resistance to rifampicin, a review. *J. Antibiot.* 67 625–630. 10.1038/ja.2014.107 25118103

[B80] Gordon D. M. O’Brien C. L. (2006). Bacteriocin diversity and the frequency of multiple bacteriocin production in *Escherichia coli*. *Microbiology* 152 3239–3244. 10.1099/mic.0.28690-0 17074895

[B81] Gordon D. M. Oliver E. Littlefield-Wyer J. (2007). “The diversity of bacteriocins in Gram-negative bacteria,” in *Bacteriocins, Ecology and Evolution*, eds Riley M. A. Chavan M. A. (Berlin: Springer).

[B82] Gratia A. (1925). Sur un remarquable exemple d’antagonisme entre deux souches de colibacille. *C. R. Soc. Biol.* 93 1041–1042.

[B83] Guijarro J. I. González-Pastor J. E. Baleux F. San Millán J. L. Castilla M. A. Rico M. (1995). Chemical structure and translation inhibition studies of the antibiotic microcin C7. *J. Biol. Chem.* 270 23520–23532. 10.1074/jbc.270.40.23520 7559516

[B84] Hantke K. (2003). Is the bacterial ferrous iron transporter FeoB a living fossil? *Trends Microbiol.* 11 192–195. 10.1016/s0966-842x(03)00100-8 12781516

[B85] Håvarstein L. S. Diep D. B. Nes I. F. (1995). A family of bacteriocin ABC transporters carry out proteolytic processing of their substrates concomitant with export. *Mol. Microbiol.* 16 229–240. 10.1111/j.1365-2958.1995.tb02295.x 7565085

[B86] Hawkey P. M. (2015). Multidrug-resistant Gram-negative bacteria, a product of globalization. *J. Hosp. Infect.* 89 241–247. 10.1016/j.jhin.2015.01.008 25737092

[B87] Heddle J. G. Blance S. J. Zamble D. B. Hollfelder F. Miller D. A. Wentzell L. M. (2001). The antibiotic microcin B17 is a DNA gyrase poison, characterisation of the mode of inhibition. *J. Mol. Biol.* 307 1223–1234. 10.1006/jmbi.2001.4562 11292337

[B88] Hegemann J. D. Zimmermann M. Zhu S. Klug D. Marahiel M. A. (2013). Lasso peptides from *proteobacteria*. Genome mining employing heterologous expression and mass spectrometry. *Biopolymers* 100 527–542. 10.1002/bip.22326 23897438

[B89] Hetz C. Bono M. R. Barros L. F. Lagos R. (2002). Microcin E492, a channel-forming bacteriocin from *Klebsiella pneumoniae*, induces apoptosis in some human cell lines. *Proc. Natl. Acad. Sci. U.S.A.* 99 2696–2701. 10.1073/pnas.052709699 11880624 PMC122410

[B90] Iyer S. S. Gensollen T. Gandhi A. Oh S. F. Neves J. F. Collin F. (2018). Dietary and microbial oxazoles induce intestinal inflammation by modulating aryl hydrocarbon receptor responses. *Cell* 173 1123–1134.e11. 10.1016/j.cell.2018.04.037 29775592 PMC6119676

[B91] Jacoby G. A. Corcoran M. A. Hooper D. C. (2015). Protective effect of Qnr on agents other than quinolones that target DNA gyrase. *Antimicrob. Agents Chemother.* 59 6689–6695. 10.1128/aac.01292-15 26239981 PMC4604369

[B92] Jacoby G. A. Mills D. M. Chow N. (2004). Role of beta-lactamases and porins in resistance to ertapenem and other beta-lactams in *Klebsiella pneumoniae*. *Antimicrob. Agents Chemother.* 48 3203–3206. 10.1128/aac.48.8.3203-3206.2004 15273152 PMC478483

[B93] James R. Lazdunski C. Pattus F. (2013). *Bacteriocins, Microcins and Lantibiotics.* Berlin: Springer.

[B94] Jansen K. B. Inns P. G. Housden N. G. Hopper J. T. S. Kaminska R. Lee S. (2020). Bifurcated binding of the OmpF receptor underpins import of the bacteriocin colicin N into *Escherichia coli*. *J. Biol. Chem.* 295 9147–9156. 10.1074/jbc.RA120.013508 32398259 PMC7335789

[B95] Jeanteur D. Schirmer T. Fourel D. Simonet V. Rummel G. Widmer C. (1994). Structural and functional alterations of a colicin-resistant mutant of OmpF porin from *Escherichia coli*. *Proc. Natl. Acad. Sci. U.S.A.* 91 10675–10679. 10.1073/pnas.91.22.10675 7524100 PMC45084

[B96] Kaeriyama M. Machida K. Kitakaze A. Wang H. Lao Q. Fukamachi T. (2006). OmpC and OmpF are required for growth under hyperosmotic stress above pH 8 in *Escherichia coli*. *Lett. Appl. Microbiol.* 42 195–201. 10.1111/j.1472-765X.2006.01845.x 16478504

[B97] Kapoor G. Saigal S. Elongavan A. (2017). Action and resistance mechanisms of antibiotics, A guide for clinicians. *J. Anaesthesiol. Clin. Pharmacol.* 33 300–305. 10.4103/joacp.JOACP_349_15 29109626 PMC5672523

[B98] Kaur K. Tarassova O. Dangeti R. V. Azmi S. Wishart D. McMullen L. (2016). Characterization of a highly potent antimicrobial peptide microcin N from uropathogenic *Escherichia coli*. *FEMS Microbiol. Lett.* 363:fnw095. 10.1093/femsle/fnw095 27190283

[B99] Kazakov T. Kuznedelov K. Semenova E. Mukhamedyarov D. Datsenko K. A. Metlitskaya A. (2014). The RimL transacetylase provides resistance to translation inhibitor microcin C. *J. Bacteriol* 196 3377–3385. 10.1128/jb.01584-14 25002546 PMC4187662

[B100] Kazakov T. Vondenhoff G. H. Datsenko K. A. Novikova M. Metlitskaya A. Wanner B. L. (2008). *Escherichia coli* peptidase A, B, or N can process translation inhibitor microcin C. *J. Bacteriol.* 190 2607–2610. 10.1128/jb.01956-07 18223070 PMC2293190

[B101] Kettles R. A. Tschowri N. Lyons K. J. Sharma P. Hengge R. Webber M. A. (2019). The *Escherichia coli* MarA protein regulates the ycgZ-ymgABC operon to inhibit biofilm formation. *Mol. Microbiol.* 112 1609–1625. 10.1111/mmi.14386 31518447 PMC6900184

[B102] Kintses B. Méhi O. Ari E. Számel M. Györkei A. Jangir P. K. (2019). Phylogenetic barriers to horizontal transfer of antimicrobial peptide resistance genes in the human gut microbiota. *Nat. Microbiol.* 4 447–458. 10.1038/s41564-018-0313-5 30559406 PMC6387620

[B103] Klaenhammer T. R. (1988). Bacteriocins of lactic acid bacteria. *Biochimie* 70 337–349. 10.1016/0300-9084(88)90206-4 3139051

[B104] Knappe T. A. Linne U. Zirah S. Rebuffat S. Xie X. Marahiel M. A. (2008). Isolation and structural characterization of capistruin, a lasso peptide predicted from the genome sequence of *Burkholderia thailandensis* E264. *J. Am. Chem. Soc.* 130 11446–11454. 10.1021/ja802966g 18671394

[B105] Kramer J. Özkaya Ö Kümmerli R. (2019). Bacterial siderophores in community and host interactions. *Nat. Rev. Microbiol.* 18 152–163. 10.1038/s41579-019-0284-4 31748738 PMC7116523

[B106] Krause K. M. Serio A. W. Kane T. R. Connolly L. E. (2016). Aminoglycosides. An overview. *Cold Spring Harb. Perspect. Med.* 6:a027029. 10.1101/cshperspect.a027029 27252397 PMC4888811

[B107] Krewulak K. D. Vogel H. J. (2008). Structural biology of bacterial iron uptake. *Biochim. Biophys. Acta* 1778 1781–1804. 10.1016/j.bbamem.2007.07.026 17916327

[B108] Kuznedelov K. Semenova E. Knappe T. A. Mukhamedyarov D. Srivastava A. Chatterjee S. (2011). The antibacterial threaded-lasso peptide capistruin inhibits bacterial RNA polymerase. *J. Mol. Biol.* 412 842–848. 10.1016/j.jmb.2011.02.060 21396375 PMC3143284

[B109] Kuznetsova M. V. Gizatullina J. S. Nesterova L. Y. Starčič Erjavec M. (2020). *Escherichia coli* isolated from cases of colibacillosis in Russian poultry farms (Perm Krai), Sensitivity to antibiotics and bacteriocins. *Microorganisms* 8:741. 10.3390/microorganisms8050741 32429211 PMC7285186

[B110] Lagos R. Tello M. Mercado G. Garcia V. Monasterio O. (2009). Antibacterial and antitumorigenic properties of microcin E492, a pore-forming bacteriocin. *Curr. Pharm. Biotechnol.* 10 74–85. 10.2174/138920109787048643 19149591

[B111] Lagos R. Wilkens M. Vergara C. Cecchi X. Monasterio O. (1993). Microcin E492 forms ion channels in phospholipid bilayer membrane. *FEBS Lett.* 321 145–148. 10.1016/0014-5793(93)80096-d 7682973

[B112] Laviña M. Gaggero C. Moreno F. (1990). Microcin H47, a chromosome-encoded microcin antibiotic of *Escherichia coli*. *J. Bacteriol.* 172 6585–6588. 10.1128/jb.172.11.6585-6588.1990 2228975 PMC526850

[B113] Laviña M. Pugsley A. P. Moreno F. (1986). Identification, mapping, cloning and characterization of a gene (sbmA) required for microcin B17 action on *Escherichia coli* K12. *J. Gen. Microbiol.* 132 1685–1693. 10.1099/00221287-132-6-1685 3543211

[B114] Lazdunski C. J. Bouveret E. Rigal A. Journet L. Lloubès R. Bénédetti H. (1998). Colicin import into *Escherichia coli* cells. *J. Bacteriol.* 180 4993–5002. 10.1128/JB.180.19.4993-5002.1998 9748429 PMC107532

[B115] Lewis K. Epstein S. D’Onofrio A. Ling L. L. (2010). Uncultured microorganisms as a source of secondary metabolites. *J. Antibiot.* 63 468–476. 10.1038/ja.2010.87 20648021

[B116] Li X. Z. Ma D. Livermore D. M. Nikaido H. (1994). Role of efflux pump(s) in intrinsic resistance of *Pseudomonas aeruginosa*, active efflux as a contributing factor to beta-lactam resistance. *Antimicrob. Agents Chemother.* 38 1742–1752. 10.1128/aac.38.8.1742 7986004 PMC284631

[B117] Li X. Z. Plesiat P. Nikaido H. (2015). The challenge of efflux-mediated antibiotic resistance in Gram-negative bacteria. *Clin. Microbiol. Rev.* 28 337–418. 10.1128/cmr.00117-14 25788514 PMC4402952

[B118] Li Y. Rebuffat S. (2020). The manifold roles of microbial ribosomal peptide-based natural products in physiology and ecology. *J. Biol. Chem.* 295 34–54. 10.1074/jbc.REV119.006545 31784450 PMC6952617

[B119] Li Y.-M. Milne J. C. Madison L. L. Kolter R. Walsh C. T. (1996). From peptide precursors to oxazole and thiazole-containing peptide antibiotics, microcin B17 synthase. *Science* 274 1188–1193. 10.1126/science.274.5290.1188 8895467

[B120] Lopez F. E. Vincent P. A. Zenoff A. M. Salomón R. A. Farias R. N. (2007). Efficacy of microcin J25 in biomatrices and in a mouse model of *Salmonella* infection. *J. Antimic. Chem.* 59 676–680. 10.1093/jac/dkm009 17353221

[B121] Lu S. Y. Graça T. Avillan J. J. Zhao Z. Call D. R. (2019). Microcin PDI inhibits antibiotic-resistant strains of *Escherichia coli* and *Shigella* through a mechanism of membrane disruption and protection by homotrimer self-immunity. *Appl. Environ. Microbiol.* 85:e0371-19. 10.1128/aem.00371-19 30902857 PMC6532030

[B122] MacVane S. H. (2017). Antimicrobial resistance in the intensive care unit, A focus on Gram-negative bacterial infections. *J. Intensive Care Med.* 32 25–37. 10.1177/0885066615619895 26772199

[B123] Maksimov M. O. Pelczer I. Link A. J. (2012). Precursor-centric genome-mining approach for lasso peptide discovery. *Proc. Natl. Acad. Sci. U.S.A.* 109 15223–15228. 10.1073/pnas.1208978109 22949633 PMC3458324

[B124] Marcoleta A. Marin M. Mercado G. Valpuesta J. M. Monasterio O. Lagos R. (2013). Microcin E492 amyloid formation is retarded by posttranslational modification. *J. Bacteriol.* 195 3995–4004. 10.1128/jb.00564-13 23836864 PMC3754591

[B125] Martin-Gómez H. Jorba M. Albericio F. Viñas M. Tulla-Puche J. (2019). Chemical Modification of microcin J25 reveals new insights on the stereospecific requirements for antimicrobial activity. *Int. J. Mol. Sci.* 20:5152. 10.3390/ijms20205152 31627419 PMC6829517

[B126] Massip C. Oswald E. (2020). Siderophore-microcins in *Escherichia coli*, determinants of digestive colonization, the first step toward virulence. *Front. Cell Infect. Microbiol.* 10:381 10.3389/fcimb.2020.00381 PMC7472721 32974212

[B127] Mathavan I. Zirah S. Mehmood S. Choudhury H. G. Goulard C. Li Y. (2014). Structural basis for hijacking siderophore receptors by antimicrobial lasso peptides. *Nat. Chem. Biol.* 10 340–342. 10.1038/nchembio.1499 24705590 PMC3992131

[B128] Mathur H. Field D. Rea M. C. Cotter P. D. Hill C. Ross R. P. (2017). Bacteriocin-antimicrobial synergy, a medical and food perspective. *Front. Microbiol.* 8:1205. 10.3389/fmicb.2017.01205 28706513 PMC5489601

[B129] McIntosh J. A. Donia M. S. Schmidt E. W. (2009). Ribosomal peptide natural products, bridging the ribosomal and nonribosomal worlds. *Nat. Prod. Rep.* 26 537–559. 10.1039/b714132g 19642421 PMC2975598

[B130] McIntosh J. A. Schmidt E. W. (2010). Marine molecular machines, heterocyclization in cyanobactin biosynthesis. *Chembiochem* 11 1413–1421. 10.1002/cbic.201000196 20540059 PMC3397156

[B131] Melby J. O. Nard N. J. Mitchell D. A. (2011). Thiazole/oxazole-modified microcins, complex natural products from ribosomal templates. *Curr. Opin. Chem. Biol.* 15 369–378. 10.1016/j.cbpa.2011.02.027 21429787 PMC3947797

[B132] Metelev M. Arseniev A. Bushin L. B. Kuznedelov K. Artamonova T. O. Kondratenko R. (2017a). Acinetodin and klebsidin, RNA polymerase targeting lasso peptides produced by human isolates of *Acinetobacter gyllenbergii* and *Klebsiella pneumoniae*. *ACS Chem. Biol.* 12 814–824. 10.1021/acschembio.6b01154 28106375

[B133] Metelev M. Osterman I. A. Ghilarov D. Khabibullina N. F. Yakimov A. Shabalin K. (2017b). Klebsazolicin inhibits 70S ribosome by obstructing the peptide exit tunnel. *Nat. Chem. Biol.* 13 1129–1136. 10.1038/nchembio.2462 28846667 PMC5701663

[B134] Metelev M. Serebryakova M. Ghilarov D. Zhao Y. Severinov K. (2013). Structure of microcin B-like compounds produced by *Pseudomonas syringae* and species specificity of their antibacterial action. *J. Bacteriol.* 195 4129–4137. 10.1128/jb.00665-13 23852863 PMC3754757

[B135] Metlitskaya A. Kazakov T. Kommer A. Pavlova O. Praetorius-Ibba M. Ibba M. (2006). Aspartyl-tRNA synthetase is the target of peptide nucleotide antibiotic microcin C. *J. Biol. Chem.* 281 18033–18042. 10.1074/jbc.M513174200 16574659

[B136] Mills S. Ross R. P. Hill C. (2017). Bacteriocins and bacteriophage; a narrow-minded approach to food and gut microbiology. *FEMS Microbiol. Rev.* 41 S129–S153. 10.1093/femsre/fux022 28830091

[B137] Mitchell D. A. Lee S. W. Pence M. A. Markley A. L. Limm J. D. Nizet V. (2009). Structural and functional dissection of the heterocyclic peptide cytotoxin streptolysin S. *J. Biol. Chem.* 284 13004–13012. 10.1074/jbc.M900802200 19286651 PMC2676033

[B138] Montalbán-López M. Scott T. A. Ramesh S. I. Rahman R. van Heel A. J. Viel J. H. (2020). New developments in RiPP discovery, enzymology and engineering. *Nat. Prod. Rep.* 10.1039/d0np00027b [Epub ahead of print]. 32935693 PMC7864896

[B139] Morin N. Lanneluc I. Connil N. Cottenceau M. Pons A. M. Sablé S. (2011). Mechanism of bactericidal activity of microcin L in *Escherichia coli* and *Salmonella enterica*. *Antimicrob. Agents Chemother.* 55 997–1007. 10.1128/aac.01217-10 21189348 PMC3067116

[B140] Mukhopadhyay J. Sineva E. Knight J. Levy R. M. Ebright R. H. (2004). Antibacterial peptide microcin J25 inhibits transcription by binding within and obstructing the RNA polymerase secondary channel. *Mol. Cell.* 14 739–751. 10.1016/j.molcel.2004.06.010 15200952 PMC2754415

[B141] Naimi S. Zirah S. Taher M. B. Theolier J. E. Fernandez B. Rebuffat S. F. (2020). Microcin J25 exhibits inhibitory activity against *Salmonella* Newport in continuous fermentation model mimicking swine colonic conditions. *Front. Microbiol.* 11:988. 10.3389/fmicb.2020.00988 32528437 PMC7262971

[B142] Nolan E. M. Walsh C. T. (2008). Investigations of the MceIJ-catalyzed posttranslational modification of the microcin E492 C-terminus, linkage of ribosomal and nonribosomal peptides to form “trojan horse” antibiotics. *Biochemistry* 47 9289–9299. 10.1021/bi800826j 18690711

[B143] Novikova M. Kazakov T. Vondenhoff G. H. Semenova E. Rozenski J. Metlytskaya A. (2010). MccE provides resistance to protein synthesis inhibitor microcin C by acetylating the processed form of the antibiotic. *J. Biol. Chem.* 285 12662–12669. 10.1074/jbc.M109.080192 20159968 PMC2857085

[B144] Novikova M. Metlitskaya A. Datsenko K. Kazakov T. Kazakov A. Wanner B. (2007). The *Escherichia coli* Yej transporter is required for the uptake of translation inhibitor microcin C. *J. Bacteriol.* 189 8361–8365. 10.1128/jb.01028-07 17873039 PMC2168686

[B145] O’Brien G. J. (1996). *Molecular Analysis of Microcin 24, Genetics, Secretion and Mode of Action of a Novel Microcin.* Ph. D thesis, University of Canterbury, Christchurch.

[B146] O’Brien G. J. Mahanty H. K. (1994). Colicin 24, a new plasmid-borne colicin from a uropathogenic strain of *Escherichia coli*. *Plasmid* 31 288–296. 10.1006/plas.1994.1030 8058821

[B147] Oyama L. B. Girdwood S. E. Cookson A. R. Fernandez-Fuentes N. Privé F. Vallin H. E. (2017). The rumen microbiome, an underexplored resource for novel antimicrobial discovery. *NPJ Biofilms Microb.* 3:33. 10.1038/s41522-017-0042-1 29214045 PMC5711939

[B148] Pagnout C. Sohm B. Razafitianamaharavo A. Caillet C. Offroy M. Leduc M. (2019). Pleiotropic effects of *rfa*-gene mutations on *Escherichia coli* envelope properties. *Sci. Rep.* 9:9696. 10.1038/s41598-019-46100-3 31273247 PMC6609704

[B149] Palmer J. D. Mortzfeld B. M. Piattelli E. Silby M. W. McCormick B. A. Bucci V. (2020). Microcin H47, A Class IIb microcin with potent activity against multidrug resistant *Enterobacteriaceae*. *ACS Infect. Dis.* 6 672–679. 10.1021/acsinfecdis.9b00302 32096972

[B150] Palmer J. D. Piattelli E. McCormick B. A. Silby M. W. Brigham C. J. Bucci V. (2018). Engineered probiotic for the inhibition of *Salmonella* via tetrathionate-induced production of microcin H47. *ACS Infect. Dis.* 4 39–45. 10.1021/acsinfecdis.7b00114 28918634 PMC5766358

[B151] Pärnänen K. Karkman A. Hultman J. Lyra C. Bengtsson-Palme J. Larsson D. G. J. (2018). Maternal gut and breast milk microbiota affect infant gut antibiotic resistome and mobile genetic elements. *Nat. Commun.* 9:3891. 10.1038/s41467-018-06393-w 30250208 PMC6155145

[B152] Patzer S. I. Baquero M. R. Bravo D. Moreno F. Hantke K. (2003). The colicin G, H and X determinants encode microcins M and H47, which might utilize the catecholate siderophore receptors FepA, Cir, Fiu and IroN. *Microbiology* 149 2557–2570. 10.1099/mic.0.26396-0 12949180

[B153] Pfeifer Y. Cullik A. Witte W. (2010). Resistance to cephalosporins and carbapenems in Gram-negative bacterial pathogens. *Int. J. Med. Microbiol.* 300 371–379. 10.1016/j.ijmm.2010.04.005 20537585

[B154] Poey M. E. Azpiroz M. F. Laviña M. (2006). Comparative analysis of chromosome-encoded microcins. *Antimicrob. Agents Chemother.* 50 1411–1418. 10.1128/aac.50.4.1411-1418.2006 16569859 PMC1426990

[B155] Pomares M. F. Delgado M. A. Corbalan N. S. Farias R. N. Vincent P. A. (2010). Sensitization of microcin J25-resistant strains by a membrane-permeabilizing peptide. *Appl. Environ. Microbiol.* 76 6837–6842. 10.1128/aem.00307-10 20802073 PMC2953009

[B156] Pons A. M. Delalande F. Duarte M. Benoit S. Lanneluc I. Sablé S. (2004). Genetic analysis and complete primary structure of microcin L. *Antimicrob. Agents Chemother.* 48 505–513. 10.1128/aac.48.2.505-513.2004 14742202 PMC321509

[B157] Pugsley A. P. Moreno F. De Lorenzo V. (1986). Microcin-E492-insensitive mutants of *Escherichia coli* K12. *J. Gen. Microbiol.* 132 3253–3259. 10.1099/00221287-132-12-3253 3309133

[B158] Ramirez M. S. Tolmasky M. E. (2010). Aminoglycoside modifying enzymes. *Drug Resist. Updat.* 13 151–171. 10.1016/j.drup.2010.08.003 20833577 PMC2992599

[B159] Ran R. Zeng H. Zhao D. Liu R. Xu X. (2017). The novel property of heptapeptide of microcin C7 in affecting the cell growth of *Escherichia coli*. *Molecules* 22:432. 10.3390/molecules22030432 28282893 PMC6155343

[B160] Rebuffat S. (2012). Microcins in action, amazing defence strategies of *Enterobacteria*. *Biochem. Soc. Trans.* 40 1456–1462. 10.1042/bst20120183 23176498

[B161] Rebuffat S. Blond A. Destoumieux-Garzón D. Goulard C. Peduzzi J. (2004). Microcin J25, from the macrocyclic to the lasso structure, implications for biosynthetic, evolutionary and biotechnological perspectives. *Curr. Protein Pept. Sci.* 5 383–391. 10.2174/1389203043379611 15544533

[B162] Rintoul M. R. de Arcuri B. F. Salomón R. A. Farias R. N. Morero R. D. (2001). The antibacterial action of microcin J25, evidence for disruption of cytoplasmic membrane energization in *Salmonella* Newport. *FEMS Microbiol. Lett.* 204 265–270. 10.1111/j.1574-6968.2001.tb10895.x 11731133

[B163] Rodriguez E. Laviña M. (2003). The proton channel is the minimal structure of ATP synthase necessary and sufficient for microcin H47 antibiotic action. *Antimicrob. Agents Chemother.* 47 181–187. 10.1128/aac.47.1.181-187.2003 12499189 PMC148971

[B164] Rosengren K. J. Clark R. J. Daly N. L. Göransson U. Jones A. Craik D. J. (2003). Microcin J25 has a threaded sidechain-to-backbone ring structure and not a head-to-tail cyclized backbone. *J. Am. Chem. Soc.* 125 12464–12474. 10.1021/ja0367703 14531690

[B165] Roy R. S. Kelleher N. L. Milne J. C. Walsh C. T. (1999). In vivo processing and antibiotic activity of microcin B17 analogs with varying ring content and altered bisheterocyclic sites. *Chem. Biol.* 6 305–318. 10.1016/s1074-5521(99)80076-3 10322125

[B166] Sablé S. Duarte M. Bravo D. Lanneluc I. Pons A. M. Cottenceau G. (2003). Wild-type *Escherichia coli* producing microcins B17, D93, J25, and L; cloning of genes for microcin L production and immunity. *Can. J. Microbiol.* 49 357–361. 10.1139/w03-047 12897830

[B167] Sablé S. Pons A. M. Gendron-Gaillard S. Cottenceau G. (2000). Antibacterial activity evaluation of microcin J25 against diarrheagenic *Escherichia coli*. *Appl. Environ. Microbiol.* 66 4595–4597. 10.1128/aem.66.10.4595-4597.2000 11010926 PMC92352

[B168] Sabnis A. Klöckner A. Becce M. Evans L. E. Furniss R. C. D. Mavridou D. A. I. (2019). Colistin kills bacteria by targeting lipopolysaccharide in the cytoplasmic membrane. *bioRxiv* [Preprint], 10.1101/479618 PMC8096433 33821795

[B169] Salah Ud-Din A. I. Tikhomirova A. Roujeinikova A. (2016). Structure and functional diversity of GCN5-related N-acetyltransferases (GNAT). *Int. J. Mol. Sci.* 17:1018. 10.3390/ijms17071018 27367672 PMC4964394

[B170] Salomón R. A. Farias R. N. (1992). Microcin 25, a novel antimicrobial peptide produced by *Escherichia coli*. *J. Bacteriol.* 174 7428–7435. 10.1128/jb.174.22.7428-7435.1992 1429464 PMC207439

[B171] Salomón R. A. Farías R. N. (1993). The FhuA protein is involved in microcin 25 uptake. *J. Bacteriol.* 175 7741–7742. 10.1128/jb.175.23.7741-7742.1993 8244949 PMC206939

[B172] Sassone-Corsi M. Nuccio S. P. Liu H. Hernandez D. Vu C. T. Takahashi A. A. (2016). Microcins mediate competition among *Enterobacteriaceae* in the inflamed gut. *Nature* 540 280–283. 10.1038/nature20557 27798599 PMC5145735

[B173] Sato M. Machida K. Arikado E. Saito H. Kakegawa T. Kobayashi H. (2000). Expression of outer membrane proteins in *Escherichia coli* growing at acid pH. *Appl. Environ. Microbiol.* 66 943–947. 10.1128/aem.66.3.943-947.2000 10698756 PMC91927

[B174] Sawa T. Kooguchi K. Moriyama K. (2020). Molecular diversity of extended-spectrum beta-lactamases and carbapenemases, and antimicrobial resistance. *J. Intens. Care* 8:13. 10.1186/s40560-020-0429-6 32015881 PMC6988205

[B175] Schindler P. R. Teuber M. (1975). Action of polymyxin B on bacterial membranes, morphological changes in the cytoplasm and in the outer membrane of *Salmonella typhimurium* and *Escherichia coli* B. *Antimicrob. Agents Chemother.* 8 95–104. 10.1128/aac.8.1.95 169730 PMC429267

[B176] Semenova E. Yuzenkova Y. Peduzzi J. Rebuffat S. Severinov K. (2005). Structure-activity analysis of microcinJ25, distinct parts of the threaded lasso molecule are responsible for interaction with bacterial RNA polymerase. *J. Bacteriol.* 187 3859–3863. 10.1128/jb.187.11.3859-3863.2005 15901712 PMC1112051

[B177] Severinov K. Nair S. K. (2012). Microcin C, biosynthesis and mechanisms of bacterial resistance. *Future Microbiol.* 7 281–289. 10.2217/fmb.11.148 22324995 PMC3350762

[B178] Sharma P. Haycocks J. R. J. Middlemiss A. D. Kettles R. A. Sellars L. E. Ricci V. (2017). The multiple antibiotic resistance operon of enteric bacteria controls DNA repair and outer membrane integrity. *Nat. Commun.* 8:1444. 10.1038/s41467-017-01405-7 29133912 PMC5684230

[B179] Shkundina I. Serebryakova M. Severinov K. (2014). The C-terminal part of microcin B is crucial for DNA gyrase inhibition and antibiotic uptake by sensitive cells. *J. Bacteriol.* 196 1759–1767. 10.1128/jb.00015-14 24563033 PMC3993328

[B180] Sikandar A. Koehnke J. (2019). The role of protein-protein interactions in the biosynthesis of ribosomally synthesized and post-translationally modified peptides. *Nat. Prod. Rep.* 36 1576–1588. 10.1039/c8np00064f 30920567

[B181] Smale S. T. (2010). Chloramphenicol acetyltransferase assay. *Cold Spring Harb. Protoc.* 2010:pdb.prot5422. 10.1101/pdb.prot5422 20439409

[B182] Smani Y. Fàbrega A. Roca I. ánchez-Encinales V. S. Vila J. Pachón J. (2014). Role of OmpA in the multidrug resistance phenotype of *Acinetobacter baumannii*. *Antimicrob. Agents Chemother.* 58 1806–1808. 10.1128/aac.02101-13 24379205 PMC3957889

[B183] Snyder A. B. Worobo R. W. (2014). Chemical and genetic characterization of bacteriocins, antimicrobial peptides for food safety. *J. Sci. Food Agric.* 94 28–44. 10.1002/jsfa.6293 23818338

[B184] Soudy R. Wang L. Kaur K. (2012). Synthetic peptides derived from the sequence of a lasso peptide microcin J25 show antibacterial activity. *Bioorg. Med. Chem.* 20 1794–1800. 10.1016/j.bmc.2011.12.061 22304849

[B185] Sun J. Liao X. P. D’Souza A. W. Boolchandani M. Li S. H. Cheng K. (2020). Environmental remodeling of human gut microbiota and antibiotic resistome in livestock farms. *Nat. Commun.* 11:1427. 10.1038/s41467-020-15222-y 32188862 PMC7080799

[B186] Temiakov D. Zenkin N. Vassylyeva M. N. Perederina A. Tahirov T. H. Kashkina E. (2005). Structural basis of transcription inhibition by antibiotic streptolydigin. *Mol. Cell.* 19 655–666. 10.1016/j.molcel.2005.07.020 16167380

[B187] Thomas X. Destoumieux-Garzón D. Peduzzi J. Afonso C. Blond A. Birlirakis N. (2004). Siderophore peptide, a new type of post-translationally modified antibacterial peptide with potent activity. *J. Biol. Chem.* 279 28233–28242. 10.1074/jbc.M400228200 15102848

[B188] Tietz J. I. Schwalen C. J. Patel P. S. Maxson T. Blair P. M. Tai H.-C. (2017). A new genome-mining tool redefines the lasso peptide biosynthetic landscape. *Nat. Chem. Biol.* 13 470–478. 10.1038/nchembio.2319 28244986 PMC5391289

[B189] Tran J. H. Jacoby G. A. (2002). Mechanism of plasmid-mediated quinolone resistance. *Proc. Natl. Acad. Sci. U.S.A.* 99 5638–5642. 10.1073/pnas.082092899 11943863 PMC122823

[B190] Travin D. Y. Bikmetov D. Severinov K. (2020). Translation-targeting RiPPs and where to find them. *Front. Genet.* 11:226. 10.3389/fgene.2020.00226 32296456 PMC7136475

[B191] Travin D. Y. Watson Z. L. Metelev M. Ward F. R. I Osterman A. I Khven M. (2019). Structure of ribosome-bound azole-modified peptide phazolicin rationalizes its species-specific mode of bacterial translation inhibition. *Nat. Commun.* 10:4563. 10.1038/s41467-019-12589-5 31594941 PMC6783444

[B192] Trimble M. J. Mlynarcik P. Kolar M. Hancock R. E. (2016). Polymyxin, Alternative mechanisms of action and resistance. *Cold Spring Harb. Perspect. Med.* 6:a025288. 10.1101/cshperspect.a025288 27503996 PMC5046685

[B193] Trujillo M. Rodriguez E. Laviña M. (2001). ATP synthase is necessary for microcin H47 antibiotic action. *Antimicrob. Agents Chemother.* 45 3128–3131. 10.1128/aac.45.11.3128-3131.2001 11600367 PMC90793

[B194] Van Boeckel T. P. Brower C. Gilbert M. Grenfell B. T. Levin S. A. Robinson T. P. (2015). Global trends in antimicrobial use in food animals. *Proc. Natl. Acad. Sci. U.S.A.* 112 5649–5654. 10.1073/pnas.1503141112 25792457 PMC4426470

[B195] Van Boeckel T. P. Glennon E. E. Chen D. Gilbert M. Robinson T. P. Grenfell B. T. (2017). Reducing antimicrobial use in food animals. *Science* 357 1350–1352. 10.1126/science.aao1495 28963240 PMC6510296

[B196] Vassiliadis G. Destoumieux-Garzón D. Lombard C. Rebuffat S. Peduzzi J. (2010). Isolation and characterization of two members of the siderophore-microcin family, microcins M and H47. *Antimicrob. Agents Chemother.* 54 288–297. 10.1128/aac.00744-09 19884380 PMC2798501

[B197] Vassiliadis G. Peduzzi J. Zirah S. Thomas X. Rebuffat S. Destoumieux-Garzón D. (2007). Insight into siderophore-carrying peptide biosynthesis, enterobactin is a precursor for microcin E492 posttranslational modification. *Antimicrob. Agents Chemother.* 51 3546–3553. 10.1128/aac.00261-07 17646411 PMC2043276

[B198] Ventola C. L. (2015). The antibiotic resistance crisis, part 1, causes and threats. *P&T* 40 277–283. 25859123 PMC4378521

[B199] Vincent P. A. Delgado M. A. Farias R. N. Salomón R. A. (2004). Inhibition of *Salmonella enterica* serovars by microcin J25. *FEMS Microbiol. Lett.* 236 103–107. 10.1016/j.femsle.2004.05.027 15212798

[B200] Vizán J. L. Hernández-Chico C. del Castillo I. Moreno F. (1991). The peptide antibiotic microcin B17 induces double-strand cleavage of DNA mediated by *E. coli* DNA gyrase. *EMBO J.* 10 467–476. 10.1002/j.1460-2075.1991.tb07969.x 1846808 PMC452668

[B201] Vondenhoff G. H. Dubiley S. Severinov K. Lescrinier E. Rozenski J. Van Aerschot A. (2011). Extended targeting potential and improved synthesis of microcin C analogs as antibacterials. *Bioorg. Med. Chem.* 19 5462–5467. 10.1016/j.bmc.2011.07.052 21855353

[B202] Wang G. Li X. Wang Z. (2016). APD3, the antimicrobial peptide database as a tool for research and education. *Nucleic Acids Res.* 44 D1087–D1093. 10.1093/nar/gkv1278 26602694 PMC4702905

[B203] Wang G. Song Q. Huang S. Wang Y. Cai S. Yu H. (2020). Effect of antimicrobial peptide microcin J25 on growth performance, immune regulation, and intestinal microbiota in broiler chickens challenged with *Escherichia coli* and *Salmonella*. *Animals* 10:345. 10.3390/ani10020345 32098236 PMC7070248

[B204] Wang X. W. Zhang W. B. (2018). Chemical topology and complexity of protein architectures. *Trends Biochem. Sci.* 43 806–817. 10.1016/j.tibs.2018.07.001 30041839

[B205] Wang Y. Chen X. Hu Y. Zhu G. White A. P. Koster W. (2018). Evolution and sequence diversity of FhuA in *Salmonella* and *Escherichia*. *Infect. Immun.* 86:e00573-18. 10.1128/iai.00573-18 30150258 PMC6204707

[B206] Wester C. W. Durairaj L. Evans A. T. Schwartz D. N. Husain S. Martinez E. (2002). Antibiotic resistance, a survey of physician perceptions. *Arch. Intern. Med.* 162 2210–2216. 10.1001/archinte.162.19.2210 12390064

[B207] Wilkens M. Villanueva J. E. Cofré J. Chnaiderman J. Lagos R. (1997). Cloning and expression in *Escherichia coli* of genetic determinants for production of and immunity to microcin E492 from *Klebsiella pneumoniae*. *J. Bacteriol.* 179 4789–4794. 10.1128/jb.179.15.4789-4794.1997 9244266 PMC179325

[B208] Wilson B. R. Bogdan A. R. Miyazawa M. Hashimoto K. Tsuji Y. (2016). Siderophores in iron metabolism, from mechanism to therapy potential. *Trends Mol. Med.* 22 1077–1090. 10.1016/j.molmed.2016.10.005 27825668 PMC5135587

[B209] Wooley R. E. Gibbs P. S. Shotts E. B. Jr. (1999). Inhibition of *Salmonella* Typhimurium in the chicken intestinal tract by a transformed avirulent avian *Escherichia coli*. *Avian Dis.* 43 245–250. 10.2307/1592614 10396637

[B210] World Health Organization [WHO] (2017). *Antibiotic Resistance Fact Sheet.* Geneva: WHO.

[B211] Yagmurov E. Tsibulskaya D. Livenskyi A. Serebryakova M. Wolf Y. I. Borukhov S. (2020). Histidine-triad hydrolases provide resistance to peptide-nucleotide antibiotics. *mBio* 11:e0497-20. 10.1128/mBio.00497-20 32265328 PMC7157772

[B212] Yan K. P. Li Y. Zirah S. Goulard C. Knappe T. A. Marahiel M. A. (2012). Dissecting the maturation steps of the lasso peptide microcin J25 in vitro. *Chembiochem* 13 1046–1052. 10.1002/cbic.201200016 22488892

[B213] Yang C. C. Konisky J. (1984). Colicin V-treated *Escherichia coli* does not generate membrane potential. *J. Bacteriol.* 158 757–759. 10.1128/JB.158.2.757-759.1984 6373733 PMC215499

[B214] Yang X. Price C. W. (1995). Streptolydigin resistance can be conferred by alterations to either the beta or beta’ subunits of *Bacillus subtilis* RNA polymerase. *J. Biol. Chem.* 270 23930–23933. 10.1074/jbc.270.41.23930 7592585

[B215] Yoneyama H. Katsumata R. (2006). Antibiotic resistance in bacteria and its future for novel antibiotic development. *Biosci. Biotechnol. Biochem.* 70 1060–1075. 10.1271/bbb.70.1060 16717405

[B216] Yorgey P. Davagnino J. Kolter R. (1993). The maturation pathway of microcin B17, a peptide inhibitor of DNA gyrase. *Mol. Microbiol.* 9 897–905. 10.1111/j.1365-2958.1993.tb01747.x 8231817

[B217] Yu H. Ding X. Shang L. Zeng X. Liu H. Li N. (2018a). Protective ability of biogenic antimicrobial peptide microcin J25 against enterotoxigenic *Escherichia coli*-induced intestinal epithelial dysfunction and inflammatory responses IPEC-J2 cells. *Front. Cell Infect. Microbiol.* 8:242. 10.3389/fcimb.2018.00242 30057893 PMC6053529

[B218] Yu H. Shang L. Zeng X. Li N. Liu H. Cai S. (2018b). Risks related to high-dosage recombinant antimicrobial peptide microcin J25 in mice model, intestinal microbiota, intestinal barrier function, and immune regulation. *J. Agric. Food Chem.* 66 11301–11310. 10.1021/acs.jafc.8b03405 30298738

[B219] Yu H. Li N. Zeng X. Liu L. Wang Y. Wang G. (2019). A comprehensive antimicrobial activity evaluation of the recombinant microcin J25 against the foodborne pathogens *Salmonella* and *E. coli* O157:H7 by using a matrix of conditions. *Front. Microbiol.* 10:1954. 10.3389/fmicb.2019.01954 31507565 PMC6718478

[B220] Yu H. Wang Y. Zeng X. Cai S. Wang G. Liu L. (2020). Therapeutic administration of the recombinant antimicrobial peptide microcin J25 effectively enhances host defenses against gut inflammation and epithelial barrier injury induced by enterotoxigenic *Escherichia coli* infection. *FASEB J.* 34 1018–1037. 10.1096/fj.201901717R 31914603

[B221] Yu H. T. Ding X. L. Li N. Zhang X. Y. Zeng X. F. Wang S. (2017). Dietary supplemented antimicrobial peptide microcin J25 improves the growth performance, apparent total tract digestibility, fecal microbiota, and intestinal barrier function of weaned pigs. *J. Anim. Sci.* 95 5064–5076. 10.2527/jas2017.1494 29293710 PMC6292272

[B222] Yuzenkova J. Delgado M. Nechaev S. Savalia D. Epshtein V. Artsimovitch I. (2002). Mutations of bacterial RNA polymerase leading to resistance to microcin J25. *J. Biol. Chem.* 277 50867–50875. 10.1074/jbc.M209425200 12401787

[B223] Zhang A. Rosner J. L. Martin R. G. (2008). Transcriptional activation by MarA, SoxS and Rob of two tolC promoters using one binding site, a complex promoter configuration for tolC in *Escherichia coli*. *Mol. Microbiol.* 69 1450–1455. 10.1111/j.1365-2958.2008.06371.x 18673442 PMC2574956

[B224] Zhao Z. Eberhart L. J. Orfe L. H. Lu S. Y. Besser T. E. Call D. R. (2015). Genome-wide screening identifies six genes that are associated with susceptibility to *Escherichia coli* microcin PDI. *Appl. Environ. Microbiol.* 81 6953–6963. 10.1128/aem.01704-15 26209678 PMC4579430

[B225] Zowawi H. M. Harris P. N. Roberts M. J. Tambyah P. A. Schembri M. A. Pezzani M. D. (2015). The emerging threat of multidrug-resistant Gram-negative bacteria in urology. *Nat. Rev. Urol.* 12 570–584. 10.1038/nrurol.2015.199 26334085

[B226] Zschüttig A. Zimmermann K. Blom J. Goesmann A. Pöhlmann C. Gunzer F. (2012). Identification and characterization of microcin S, a new antibacterial peptide produced by probiotic *Escherichia coli* G3/10. *PLoS One* 7:e033351. 10.1371/journal.pone.0033351 22479389 PMC3316575

