# Supplementary material for: Bacteriocins to Thwart Bacterial Resistance in Gram Negative Bacteria
Source: Front Microbiol. 2020 Nov 9;11:586433. doi: 10.3389/fmicb.2020.586433 (PMC7680869; doi:10.3389/fmicb.2020.586433)

Bacteriocins to thwart bacterial resistance in Gram negative bacteria

Soufiane Telhig[1,2$], Laila Ben Said[1$], Séverine Zirah[2], Ismail Fliss[1] and Sylvie Rebuffat[2*]

[1] Institute of Nutrition and Functional Foods, Université Laval, G1V 0A6 QC, Canada
[2] Laboratory Molecules of Communication and Adaptation of Microorganisms, Muséum National d'Histoire Naturelle, Centre National de la Recherche Scientifique, CP 54, 57 rue Cuvier 75005, Paris, France

[$] Both authors contributed equally to the manuscript

Supplementary information

Figure S1: Precursor sequences and structures of microcins and analogs. A) Amino acid sequence alignments of the precursors of class I microcins and analogs discovered by genome mining (only analogs with activity against Gram-negative bacteria and/or against the identified intracellular target are shown, independently of the producing strain) and representative structures of the mature peptides. For McC and analogs, the gene encoded C-terminal Asn residue is converted to Asp and linked to a nucleotide, represented as a black square. Both the structures of the McC heptapeptide nucleotide secreted by the producing strains and of the final toxic entity generated by proteolytic cleavages in sensitive strains are shown; **B)** Amino acid sequence alignments of the precursors of class IIa microcins; cysteines involved in disulfide bridges are boldfaced; **C)** Amino acid sequence alignments of the precursors of class IIb siderophore microcins. The siderophore moiety (linear trimer of DHBS) at the C-terminus is shown as a black circle; its structure is shown below the alignments. Alignments were generated using Muscle (https://www.ebi.ac.uk/Tools/msa/muscle). Conserved and semi-conserved amino acids are light grey and grey shaded, respectively. For modified microcins, the amino acids that undergo PTM are shown in bold red. Red bars indicate cleavage sites between the leader peptide and core sequence, observed during the maturation. Green bar indicates cleavage in the target bacteria yielding the toxic entity for McC and analogs.

A

Class I microcins and analogs

Peptide nucleotides (McC-like)

```
Gene-derived McC              -------MKLS-----------YRN
Mature McC                    -------MKLS-----------YRD-■
Helicobacter pylori           -------MKLS-----------YRD-■
Streptococcus thermophilus    ----LMDMKGT-----------ILD-■
Lactobacillus johnsonii       -------MHRI-----------MKD-■
Synechococcus sp.             SRKTLLQPKRLDKVAKNQLWADMMD-■
                                      :               *
```

Lasso peptides (McJ25-like)

leader peptide **core peptide**

```
Capistruin      ---------MVRLLAKLLRSTIHGSNGVSLDAVSSTHGTPG----FQTPDARV-------ISRFGFN
Ubonodin        -----MKNRSTKESFEITCIGDVDVITLMQDASRATMGGDGSIAEYFNRPMHIHDWQIMDSGYYG--
Acinetodin      -----MKNLNKMFKKEQKYHKQLKVISVKGSASKMTLGGKGPIFETWVTEG----------NYYG--
Klebsidin       -----------MMQQKKNDQKKVTLKKLNKKASKVTRGSDGPIIEFFNPNG---------VMHYG--
McJ25           MIKHFHFNKLSSGKKNNVPSPAKGVIQIKKSASQLTKGGAGHVPEYFVGIGTP-------ISFYG--
Citrocin        -----------MKKQTFVPKKLVKV----GKATELTKGGVGKIIEYFIGGG---------VGRYG--
                                              .*   * *  *                      :*
```

LAPs (MccB17-like)

```
Phazolicin      MTTQILNPPQFGTEI-------EFVDA-------------GDSTVQTAATCARCDSSSRCGASGKSSGSASSIST--
Klebsazolidin   -MSKIKN--RFGSQLA------SFNSPVKKGL--------YSQSPGNCASCSNSASANCTGGLG-------------
MccB17          MELKAS---EFGVVLSVDALKLSRQSPLGVGIGGGGGGGGGGSCGGQGGGCGGC-SNGCSGGNGGSGGSGSHI----
Ps_McB          MENDYIS--EFGEVVAVDGQRSSFDRP-HTSL--------GGSCGGQGGGCGGCGGGGCSGGNGGSGGSGTSAPDHV
                   .      **  :       .   .              ..     . *. .  .  . *. *
```

McC

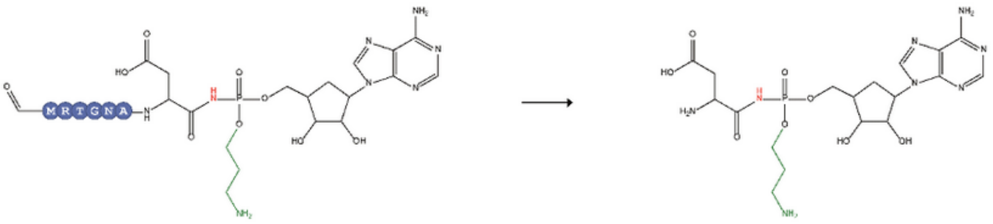

Mccj25

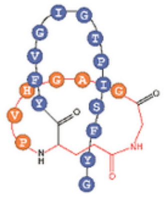

MccB17

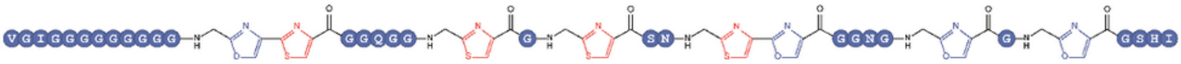

B
Class IIa microcins

```
MccN/24      ---MRELDREELNCVGGAGDPLADPNSQIVRQIMSNAAWGAAFGARGGLGGMAVGA----------AGGVTQTVLQ
MccPDI       MANIRELTLDEITLVSGG---------NANSNFEGGPRNDRSSGARNSLGRNAPTHIYSDPSTVKCANAVFSGMIG
MccS         MSNIRELSFDEIALVSGG---------NANSNYEGGGSRSRNTGARNSLGRNAPTHIYSDPSTVKCANAVFSGMVG
MccL         ---MREITLNEMNNVSGAGDVNWVDVGKTVAT-NGAGVIGGAFGA--GLCGPVCAGAFAVGSSAAVAALYDAAGNS
MccV         ---MRTLTLNELDSVSGG--ASGRDIAMAIGT-LSGQFVAGGIGA--AAGGVAGGAIYDYASTHKPNPAMSPSGLG
                :* :  :*:  *.*.                .        **  .    .

MccN/24      GAAAHMPVNVPIPKVPMGP---------------------SWNGSKG------
MccPDI       GAIKGGPIGMARGTIGGAVVGQCLSDHGSGNGSGNRGSSSSCSGNNVGGTCNR
MccS         GAIKGGPVGMTRGTIGGAVIGQCLSGGGNGNGGGNRAGSSNCSGSNVGGTCSR
MccL         NSAKQKPEGLPPEAWNYAEGRMC-----------------NWSPNNLSDVCL-
MccV         GTIKQKPEGIPSEAWNYAAGRLC-----------------NWSPNNLSDVCL-
             .:    * .:.      .                      . .  .:
```

C
Class IIb microcins

```
MccE492         MREISQKDLNLAFGAGETDPNTQLLNDLGNNMAWGAALGAPGGLG-SAALGAAGGALQTV
MccM            MRKLSENEIKQISGGDGNDGQAELIA-------IGSLAGTFISPGFGSIVGAYIGDKVHS
MccH47          MREITESQLRYISGAGGAPATSANAA--GAAAIVGALAGIPGGPL-GVVVGA--------
                **::::.::.   *..     :            *:  *   .   .  :**

MccE492         GQGLIDHGPVNVPIPVLIGPSWNGSGSGYNSATSSSGSGS-●
MccM            WATTATVSPSMSPSGIGLSSQF-GSGRGTSSASSSAGSGS-●
MccH47          -------------VSAGLTTAI-GSTVGSGSASSSAGGGS-●
                                 : .   **  * .**:**:*.**
```

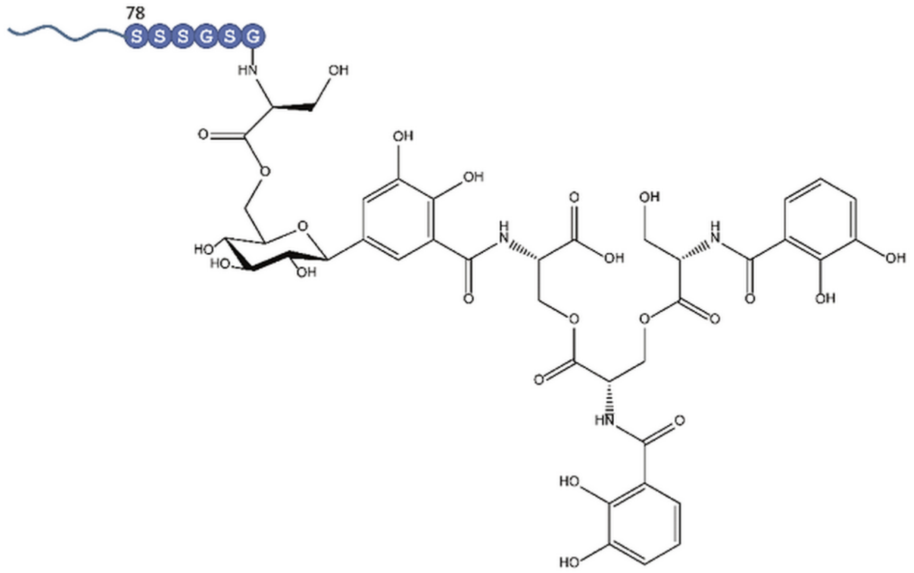

Supplement: Supplementary file 1 [file Image_1.pdf]
